# Integrating Radiomics and Computational Pathology to Predict Early Recurrence of Pancreatic Ductal Adenocarcinoma and Uncover Its Biological Basis in Tumor Microenvironment

**DOI:** 10.1002/advs.202523985

**Published:** 2026-04-13

**Authors:** Sihang Cheng, Fuze Cong, Shenbo Zhang, Rui Lv, Wenjia Zhang, Xinyi Ke, Juncheng Wu, Zhonghe Zhao, Kui Zhao, Di Dong, Ruofan Zhang, Zhengyu Jin, Max Seidensticker, Zhiwei Wang, Huanwen Wu, Xianlin Han, Nan Hong, Huadan Xue

**Affiliations:** ^1^ Department of Radiology Peking Union Medical College Hospital Chinese Academy of Medical Sciences & Peking Union Medical College Beijing China; ^2^ Department of Radiology Peking University People's Hospital Beijing China; ^3^ Department of Pathology Peking Union Medical College Hospital Chinese Academy of Medical Sciences & Peking Union Medical College Beijing China; ^4^ School of Artificial Intelligence University of Chinese Academy of Sciences Beijing China; ^5^ CAS Key Laboratory of Molecular Imaging Institute of Automation Chinese Academy of Sciences Beijing China; ^6^ Department of Radiology University Hospital LMU Munich Munich Germany; ^7^ Department of General Surgery Peking Union Medical College Hospital Chinese Academy of Medical Sciences & Peking Union Medical College Beijing China

**Keywords:** computational pathology, early recurrence, extracellular matrix remodeling, pancreatic ductal adenocarcinoma, radiomics

## Abstract

**Background**: Accurate prediction of early recurrence (ER) after radical resection remains a critical challenge in pancreatic ductal adenocarcinoma (PDAC). This study aimed to develop and validate an integrated radiomic‐pathology (Rad‐Path) model for ER prediction and to elucidate its underlying biological mechanisms.

**Methods**: A retrospective cohort of 225 PDAC patients who underwent R0 resection was included. Preoperative CT images and whole‐slide images (WSI) were collected for the extraction of radiomic features and computational pathology features. Selected features were used to develop 11 distinct machine learning models. The SHapley Additive exPlanations (SHAP) algorithm was employed to evaluate feature importance. Single‐cell RNA sequencing (scRNA‐seq) and spatial transcriptomics (ST) were performed on prospectively collected specimens.

**Results**: The final Rad‐Path model achieved AUCs of 0.851 and 0.814 in the internal and external validation cohorts, respectively. The predicted ER group was specifically linked to the enrichment of fibroblasts and pancreatic stellate cells, as well as dysregulation in extracellular matrix (ECM)‐related pathways. This finding was validated histopathologically, as predicted ER patients predominantly displayed a “reactive‐dominant” phenotype marked by abundant activated fibroblasts and ECM deposition.

**Conclusion**: Our study offers a high‐performance predictive model for ER in PDAC and establishes ECM remodeling as a key biological mechanism underlying the predictions.

## Graphical abstract

Accurate prediction of early recurrence in pancreatic ductal adenocarcinoma is vital for optimizing treatment. A novel, integrated radiomics‐pathology machine learning model successfully forecasts recurrence risks by analyzing preoperative CT images and computational pathology. This approach uniquely links predictive features to extracellular matrix remodeling and fibroblast enrichment, unveiling critical biological insights into the tumor microenvironment to guide personalized interventions.

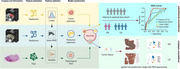

**Abbreviations**
AB: Adaptive Boosting
ALiBi: Attention with Linear Biases
AUC: Area Under the Curve
BNB: Bernoulli Naive Bayes
CECT: Contrast Enhanced Computed Tomography
CNV: Copy Number Variation
CypA: Cyclophilin A
DT: Decision Tree
ECM: Extracellular Matrix
ER: Early Recurrence
GB: Gradient Boosting
GNB: Gaussian Naive Bayes
H&E: Hematoxylin and Eosin
KNN: k‐Nearest Neighbors
LASSO: Least Absolute Shrinkage and Selection Operator
LDA: Linear Discriminant Analysis
LR: Late Recurrence
L‐R: Ligand‐Receptor
LRG: Logistic Regression
OSM: Oncostatin M
PARs: Protease‐Activated Receptors
PCA: Principal Component Analysis
PDAC: Pancreatic Ductal Adenocarcinoma
PSCs: Pancreatic Stellate Cells
Rad‐Path: Radiomics‐Pathology
RF: Random Forest
RFS: Recurrence‐Free Survival
RORA: RAR‐Related Orphan Receptor A
scRNA‐seq: Single‐Cell RNA Sequencing
SHAP: SHapley Additive exPlanations
ST: Spatial Transcriptomics
TME: Tumor Microenvironment
SVM: Support Vector Machine
UMAP: Uniform Manifold Approximation and Projection
UMI: Unique Molecular Identifier
WSIs: Whole‐Slide Images
XGB: XGBoost

## 1 Introduction

Pancreatic ductal adenocarcinoma (PDAC) is a highly aggressive malignancy, and radical resection remains the primary curative treatment [1, 2, 3]. However, early recurrence (ER)—typically defined as recurrence occurring within 6 or 12 months post‐surgery—is associated with significantly different survival outcomes compared to late recurrence (LR) [4, 5, 6, 7]. Notably, patients recurring within 6 months have a particularly poor prognosis [5]. Therefore, accurate prediction of ER within 6 months is essential to improve treatment strategies and facilitate individualized care.

A range of clinical indicators has been identified as predictors of survival following radical resection [5, 8]. In clinical practice, radiological imaging enables the identification of high‐risk features indicative of aggressive disease [2], and models derived from these imaging features demonstrate strong utility for risk stratification [9, 10]. To further enhance objectiveness, radiomics, as a non‐invasive approach, mitigates inter‐observer variability and enables the quantitative characterization of a lesion's underlying pathophysiology [11]. It has been used to predict key clinical outcomes in PDAC, including tumor behavior and ER, demonstrating promising accuracy [12, 13, 14, 15, 16]. Beyond radiomics, computational pathology represents a powerful tool for high‐throughput extraction and analysis of quantitative features from digitized pathology images, demonstrating strong performance across multiple malignancies, including pancreatic cancer [17], urothelial carcinoma [18], and gastrointestinal cancers [19]. Nonetheless, the biological basis of ER‐associated radiomic and computational pathology features in PDAC is poorly defined, limiting their interpretability for predicting recurrence.

SHapley Additive exPlanations (SHAP) enables the assessment of both global feature importance and individual variable effects [20] and has been widely applied in biomedical research [21, 22, 23]. Wu et al. developed an interpretable machine learning model to predict ER in PDAC by integrating radiomic and body composition features and employing SHAP for explanation [24]. While SHAP clarifies feature contributions, it does not directly reveal the molecular pathways behind imaging phenotypes. Single‐cell RNA sequencing (scRNA‐seq) and spatial transcriptomics (ST) provide deep insights into the tumor microenvironment (TME) and cell‐cell interactions, while precisely mapping cells to their anatomical background. Joint scRNA‐seq and ST analyses have been successfully applied to various cancers, such as bladder, lung, and colorectal cancer, revealing intratumoral heterogeneity and tumor–stromal crosstalk [25, 26, 27]. A key advantage of applying scRNA‐seq with ST in imaging‐based models is the ability to bridge macroscopic imaging findings with underlying molecular activity. Feng et al. demonstrated that a CT‐based radiomic model predicting the macrotrabecular‐massive subtype of hepatocellular carcinoma was associated with impaired humoral immunity [28]. A key limitation of previous PDAC radiomic models is that they stand apart from computational pathology and have left the biological basis of their predictions unexplored by scRNA‐seq or ST. Thus, a deeper investigation into the biological mechanisms underpinning radiomic and pathologic features associated with ER is urgently needed to improve model interpretability and clinical applicability.

To address this gap, we aim to develop and validate an integrated model for predicting ER (≤6 months) in PDAC by combining CT radiomics with computational pathology. To this end, we have prospectively collected patient samples for scRNA‐seq and ST analysis to define the molecular and microenvironmental characteristics of the predicted high‐risk subgroup. The ultimate goal of our study is to elucidate the biological basis underlying the model's predictions.

## 2 Results

**FIGURE 1:**
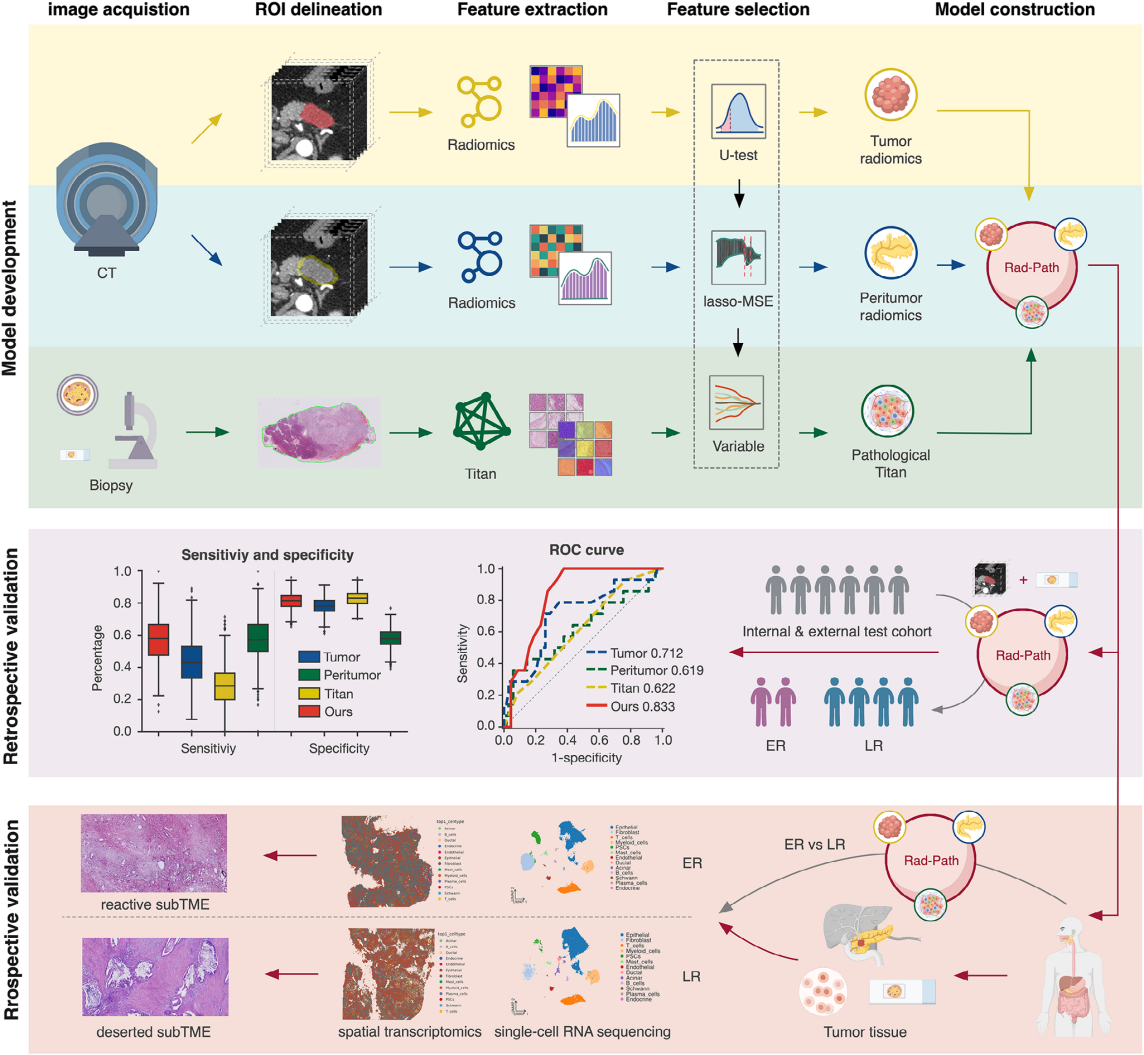
Study workflow. Pretreatment CT images and biopsy whole‐slide images were retrospectively collected and segmented for feature extraction. Following feature selection, three distinct feature sets (tumor‐region radiomics, peritumoral‐region radiomics, and pathological features) were identified and subsequently used to construct the Rad‐Path model. The performance of the model in predicting ER was evaluated in internal and external validation cohorts, with biological mechanisms finally explored through multi‐omics analysis. Abbreviations: ROI: Region of Interest; ROC: c; ER: Early Recurrence; LR: Late Recurrence.

The workflow of this study is illustrated in Figure 1.

### 2.1 Patient Characteristics

**FIGURE 2:**
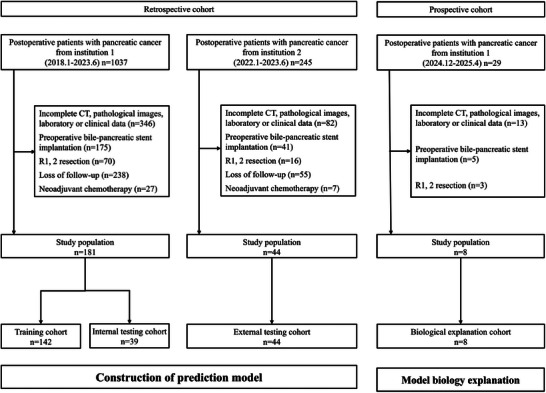
Participants inclusion and exclusion process.

The cohort comprised 225 patients (121 men and 104 women) with a median age of 62 years. Of these, 181 patients from Institution 1 (Jan 2018–Jun 2023) were randomly split into a training set (n = 142) and an internal test set (n = 39). An additional 44 patients from Institution 2 (Jan 2022–Jun 2023) served as an external test set (Figure 2). The demographic, clinical, and pathological characteristics, laboratory parameters, and survival outcomes of the patients are summarized in Table 1. Most patients denied a significant history of smoking, alcohol use, or relevant comorbidities. The median tumor diameter was 3.0 cm. Tumor localization was predominantly (55%) in the pancreatic head, and peripheral invasion was pathologically absent in 53% of patients. Baseline characteristics were well‐balanced between the training and internal validation cohorts, with no statistically significant differences (all *p* > 0.05). Recurrence‐free survival (RFS) was defined as the time from surgery to recurrence or death from any cause, whichever occurred first. The median RFS for the entire cohort was 19.40 months. A comparison was performed between the ER and LR groups across both institutions (Table S1). Recurrence distributions and chemotherapy regimens were comparable (*p* > 0.05). Since most recurrences occurred during chemotherapy, this institutional consistency in outcome distribution and treatment supports the reliability of our model.

**TABLE 1:** Descriptive Statistics of Study Variables (N = 225).

Variable	Total (n = 225)	Training cohort (n = 142)	Internal validation cohort (n = 39)	External validation cohort (n = 44)	Statistic	*p* value
**Demographic Characteristics**						
Age, years	62.00 [56.00, 69.00]	63.00 [56.25, 69.00]	61.00 [54.50, 67.00]	62.50 [57.00, 69.00]	H = 0.970, df = 2	0.62
Sex, male/female	121/104	73/69	23/16	25/19	χ^2^ = 0.908, df=2	0.64
BMI, kg/m^2^	23.11 [21.22, 24.97]	23.06 [21.26, 24.82]	24.22 [20.94, 25.50]	22.17 [21.06, 24.50]	H = 1.825, df = 2	0.40
**Clinical Characteristics**						
Smoking history, no/yes	151/74	100/42	20/19	31/13	χ^2^ = 5.356, df = 2	0.07
Drinking history, no/yes	153/72	99/43	23/16	31/13	χ^2^ = 1.775, df = 2	0.41
Diabetes, no/yes	154/71	95/47	31/8	28/16	χ^2^ = 6.720, df = 4	0.15
Hypertension, no/yes	125/100	78/64	25/14	22/22	χ^2^ = 1.726, df = 2	0.42
Hyperlipidemia, no/yes	201/24	126/16	37/2	38/6	χ^2^ = 1.716, df = 2	0.42
Pancreatitis, no/yes	196/29	121/21	37/2	38/6	χ^2^ = 12.490, df = 6	0.05
Pancreatic cancer family history, no/yes	213/12	133/9	39/0	41/3	χ^2^ = 2.673, df = 2	0.26
**Laboratory Parameters**						
LYMPH, %	27.30 [22.10, 32.60]	27.05 [21.45, 31.60]	29.70 [23.25, 33.10]	27.15 [21.70, 31.57]	H = 2.008, df = 2	0.33
MONO, %	6.30 [5.10, 7.20]	6.20 [5.10, 7.10]	5.70 [5.20, 7.15]	6.60 [5.70, 7.40]	H = 3.439, df = 2	0.179
NEUT, %	61.80 [56.30, 67.40]	61.85 [56.30, 68.58]	60.70 [53.05, 65.25]	63.30 [58.25, 67.70]	H = 4.646, df = 2	0.10
ALT, U/L	21.00 [13.00, 55.00]	19.00 [13.00, 41.75]	24.00 [12.50, 57.50]	31.50 [17.75, 140.25]	H = 7.127, df = 2	0.03*
AST, U/L	21.00 [16.00, 40.00]	19.50 [15.00, 37.75]	20.00 [16.50, 33.00]	25.00 [19.00, 72.25]	H = 6.441, df = 2	0.04*
TP, g/L	67.24 [64.00, 71.00]	67.12 [63.25, 71.00]	68.00 [65.00, 70.00]	67.12 [62.00, 71.65]	H = 0.362, df = 2	0.83
ALB, g/L	41.00 [39.00, 44.00]	41.00 [39.00, 44.00]	42.00 [40.50, 44.00]	41.00 [39.00, 44.92]	H = 0.947, df = 2	0.62
TBIL, µmol/L	13.70 [9.80, 29.90]	12.40 [9.70, 27.82]	14.40 [8.95, 24.60]	16.70 [11.38, 67.90]	H = 4.748, df = 2	0.07
GGT, U/L	26.00 [17.00, 146.00]	25.00 [16.00, 139.50]	21.00 [16.50, 84.00]	43.50 [22.00, 211.50]	H = 7.855, df = 2	0.02*
TBA, µmol/L	3.80 [2.00, 11.70]	3.55 [2.00, 9.45]	4.30 [2.25, 14.25]	6.55 [2.50, 17.70]	H = 0.895, df = 2	0.04*
GLU, mmol/L	6.20 [5.30, 7.70]	6.20 [5.40, 7.57]	5.70 [5.15, 7.60]	6.20 [5.47, 7.80]	H = 1.095, df = 2	0.58
TG, mmol/L	1.26 [0.94, 1.72]	1.21 [0.92, 1.60]	1.30 [0.99, 1.89]	1.38 [0.99, 1.85]	H = 1.951, df = 2	0.41
CRP, mg/L	1.76 [0.73, 4.86]	1.46 [0.73, 4.01]	2.01 [0.74, 5.69]	2.72 [0.67, 7.14]	H = 0.496, df = 2	0.25
CA19‐9, U/mL	124.70 [38.20, 332.00]	127.85 [38.90, 342.75]	121.00 [26.50, 299.70]	124.50 [32.70, 277.02]	H = 0.905, df = 2	0.67
CEA, µg/L	2.86 [1.80, 4.40]	3.10 [1.80, 4.38]	3.00 [1.87, 4.54]	2.50 [1.70, 4.14]	H = 1.013, df = 2	0.66
CA125, U/mL	14.30 [8.70, 21.22]	15.05 [9.75, 21.22]	10.80 [0.00, 21.76]	13.50 [7.80, 21.22]	H = 10.393, df = 2	0.12
**Pathological Characteristics**						
Max Diameter, cm	3.00 [2.20, 4.00]	3.00 [2.10, 4.00]	3.00 [2.20, 4.20]	3.00 [2.30, 3.50]	H = 1.014, df = 2	0.59
Tumor location, head/body/tail/whole	124/46/52/3	72/31/37/2	23/5/10/1	29/10/5/0	χ^2^ = 7.195, df = 6	0.30
Peripheral invasion, no/yes	119/106	79/63	19/20	21/23	χ^2^ = 1.172, df = 2	0.56
Cell differentiation, poor/moderate/well	77/138/10	50/86/6	14/21/4	13/31/0	χ^2^ = 7.563, df = 6	0.18
Lymph node metastasis, no/yes	111/114	70/72	16/23	25/19	χ^2^ = 2.063, df = 2	0.36
T stage, T1/T2/T3/T4	50/116/47/12	31/72/30/9	8/19/11/1	11/25/6/2	χ^2^ = 3.523, df = 6	0.74
N stage, N0/N1/N2	111/91/23	70/56/16	16/18/5	25/17/2	χ^2^ = 3.227, df = 4	0.52
**Survival Outcomes**						
Median RFS, months	19.40 [6.47, 35.83]	14.75 [5.49, 32.17]	19.40 [7.67, 53.53]	24.05 [9.17, 39.40]	H = 3.877, df = 2	0.14

*Note*: Data are presented as Mean ± Standard deviation or Median [25th Percentile, 75th Percentile] for continuous variables and counts for categorical variables. P‐values for continuous variables were calculated using one‐way analysis of variance (ANOVA, F statistic) or Kruskal‐Wallis H test (H statistic); for categorical variables, Pearson's chi‐square test (χ^2^) or Fisher's exact test (Fisher's exact) was used as appropriate. The corresponding test statistic is provided in the “Statistic” column. *Although a statistically significant difference (p < 0.05) was initially observed, no significant differences were found between the training cohort and internal validation cohort upon further verification. Abbreviations: χ^2^, Chi‐square statistic; df, Degrees of freedom; BMI, Body Mass Index; LYMPH, lymphocyte; MONO, monocyte; NEUT, neutrophil; ALT, alanine aminotransferase; AST, aspartate aminotransferase; TP, total protein; ALB, albumin; TBIL, total bilirubin; GGT, gamma‐glutamyl transferase; TBA, total bile acid; GLU, glucose; TG, triglycerides; CRP, C‐reactive protein; CA19‐9, Carbohydrate Antigen 19‐9; CEA, carcinoembryonic antigen; CA125, Cancer Antigen 125; RFS, recurrence‐free survival.

### 2.2 Generation of the Radiomic‐Pathology Models

**FIGURE 3:**
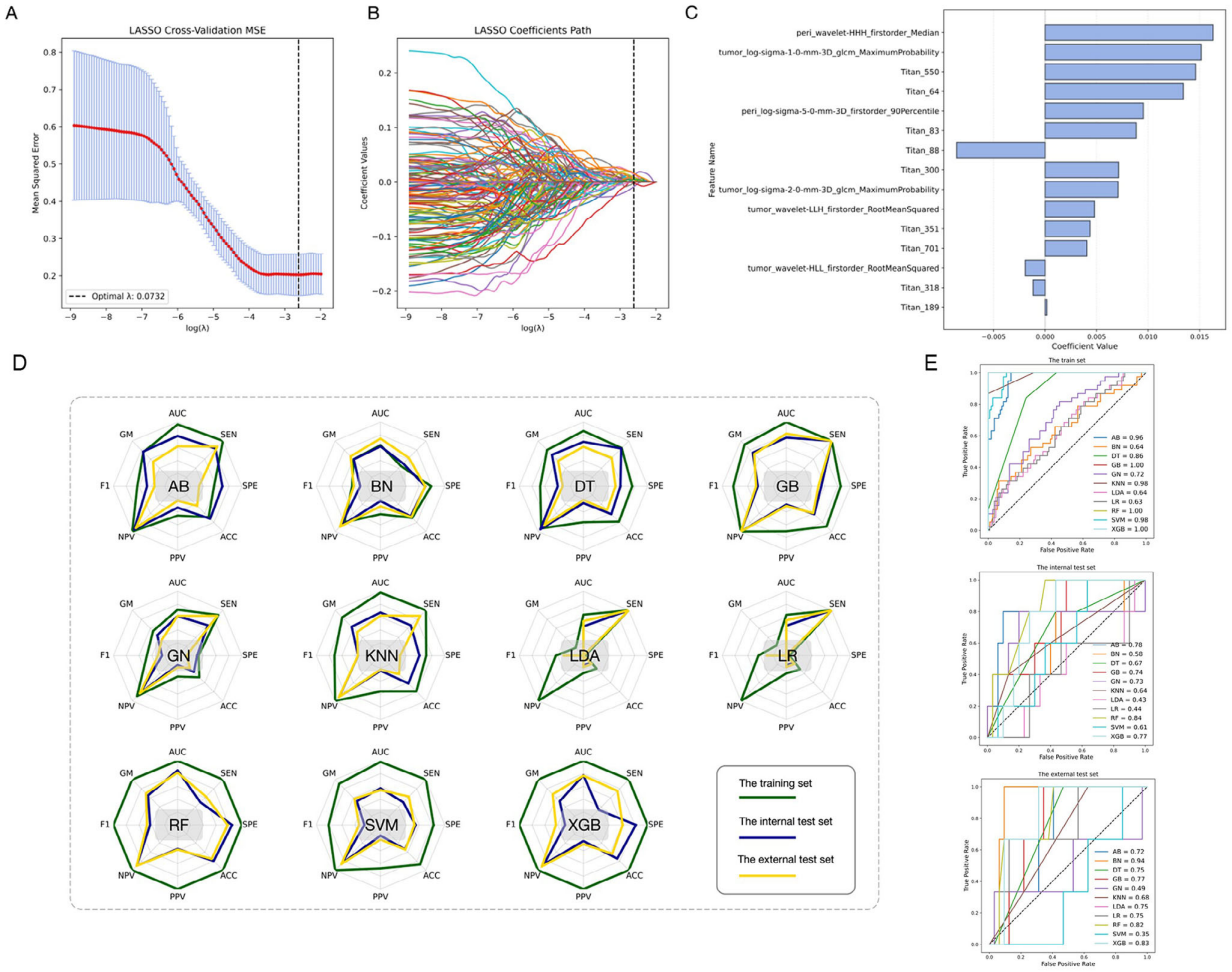
Multivariable selection of radiomic and pathological features using LASSO regression and model performance evaluation. (A) Trend of the mean square error (MSE) across different values of the penalty parameter λ during cross‐validation. The red dots indicate the average MSE values, with blue error bars representing the standard deviation. The optimal λ value, where the MSE is minimized, is marked by the black dotted line. (B) Convergence paths of feature weight coefficients as λ varies. Each line corresponds to a radiomic feature previously identified in univariable analysis. At the optimal λ = 0.0731, the MSE reaches its minimum value of 0.2022 ± 0.0558, and 15 features with non‐zero weight coefficients are retained. (C) Names and weight coefficients of the 15 selected features. (D) Radar charts displaying eight performance indicators across different datasets for all eleven machine learning models. (E) Receiver operating characteristic curve analysis illustrating the performance of the models in differentiating ER and LR patients within the training set, internal test set, and external test set. Abbreviations: LASSO: Least Absolute Shrinkage and Selection Operator; MSE: Mean Squared Error; AB: Adaptive Boosting; BNB: Bernoulli Naive Bayes; DT: Decision Tree; GB: Gradient Boosting; GN: Gaussian Naive Bayes; KNN: k‐Nearest Neighbors; LDA: Linear Discriminant Analysis; LRG: Logistic Regression; RF: Random Forest; SVM: Support Vector Machine; XGB: XG Boost; ER: Early Recurrence; LR: Late Recurrence.

In the training set, univariable analysis of 3204 features (1218 tumor, 1218 peritumoral, and 768 pathological) with *p* < 0.10 yielded 950 candidates for least absolute shrinkage and selection operator (LASSO) regression, including 361 (38%) tumor, 392 (41%) peritumoral, and 197 (21%) pathological features. The LASSO regression process is illustrated in Figure 3. Specifically, Figure 3A,B depicts the trajectory of the mean squared error (MSE) and the weight coefficients of the features across different Lambda (λ) values, respectively. The optimal λ value of 0.0731 (indicated by the black dashed lines in Figure 3A,B) was selected at the point where the MSE was minimized (0.2022 ± 0.0558), yielding a final set of 15 features with non‐zero coefficients (Figure 3C).

Based on the selected features, 11 distinct models were constructed using a range of commonly‐employed machine learning classifiers, including Adaptive Boosting (AB), Bernoulli Naive Bayes (BNB), Decision Tree (DT), Gradient Boosting (GB), Gaussian Naive Bayes (GNB), k‐Nearest Neighbors (KNN), Linear Discriminant Analysis (LDA), Logistic Regression (LRG), Random Forest (RF), Support Vector Machine (SVM), and XGBoost (XGB). Their performance is summarized in Figure 3, which includes radar charts of eight evaluation metrics and Receiver Operating Characteristic (ROC) curves for both training and test sets, with detailed results in Table 2. Analysis of the area under the curve (AUC) revealed that in the training set, the highest AUC was 1.00 (achieved by GB, RF, and XGB classifiers), while the lowest was 0.634 (LRG classifier). In the internal test set, AUC values ranged from 0.85 (RF) to 0.455 (LRG). For the external test set, the AUC peaked at 0.819 (GB) and dropped to a low of 0.538 (LDA). Comprehensive performance metrics for all radiomic‐pathology (Rad‐Path) models are available in Table S2 (training set), Table S3 (internal test set), and Table S4 (external test set).

**TABLE 2:** Performance of the Rad‐Path models on different datasets.

Model	AUC of training set	AUC of internal test set	AUC of external test set
AB	0.964 [0.934–0.985]	0.783 [0.486–0.990]	0.622 [0.452–0.777]
BNB	0.640 [0.531–0.746]	0.621 [0.343–0.853]	0.743 [0.514–0.930]
DT	0.861 [0.811–0.908]	0.692 [0.450–0.868]	0.616 [0.451–0.767]
GB	1.000 [1.000–1.000]	0.758 [0.565–0.924]	0.819 [0.689–0.927]
GN	0.716 [0.628–0.806]	0.616 [0.236–0.963]	0.618 [0.375–0.850]
KNN	0.981 [0.962–0.996]	0.672 [0.384–0.917]	0.623 [0.430–0.810]
LDA	0.636 [0.536–0.731]	0.455 [0.164–0.741]	0.538 [0.318–0.785]
LRG	0.634 [0.528–0.729]	0.465 [0.176–0.737]	0.552 [0.329–0.759]
RF	1.000 [1.000–1.000]	0.851 [0.706–0.970]	0.814 [0.682–0.931]
SVM	0.982 [0.963–0.995]	0.571 [0.300–0.815]	0.543 [0.318–0.753]
XGB	0.982 [0.963–0.995]	0.571 [0.300–0.815]	0.543 [0.318–0.753]

*Note*: Performance metrics are presented as the AUC with 95% confidence intervals in square brackets. Abbreviations: AB = Adaptive Boosting, BN = Bernoulli NB, DT = Decision Tree, GB = Gradient Boosting, GN = Gaussian NB, K Nearest Neighbors = KNN, Linear Discriminant Analysis = LDA, Logistic Regression = LRG, Random Forest = RF, SVM = Support Vector Machine, XGB = XG Boost.

The perfect performance (AUC = 1.000, sensitivity = 1.000, specificity = 1.000) achieved by several models (GB, RF, XGB) on the training set (Table S2) is a strong indicator of overfitting. This typically occurs when a model learns noise or specific patterns in the training data that do not generalize to new data. We explicitly acknowledge this as a limitation of our modeling approach. To mitigate the impact of overfitting and select the most generalizable model, our selection criterion prioritized consistent and robust performance on the independent validation cohorts, rather than the training performance. Among all classifiers, the Random Forest (RF) model demonstrated the most stable and superior performance across both the internal (AUC 0.851) and external (AUC 0.814) test sets (Table 2), despite its perfect training scores. The observed drop in sensitivity (from 1.000 to 0.500 in the internal test set) further underscores the necessity of rigorous external validation. The RF model's ensemble nature, combined with the feature selection via LASSO regression, likely contributed to its relatively better generalization compared to other models that showed greater performance degradation in validation.

**FIGURE 4:**
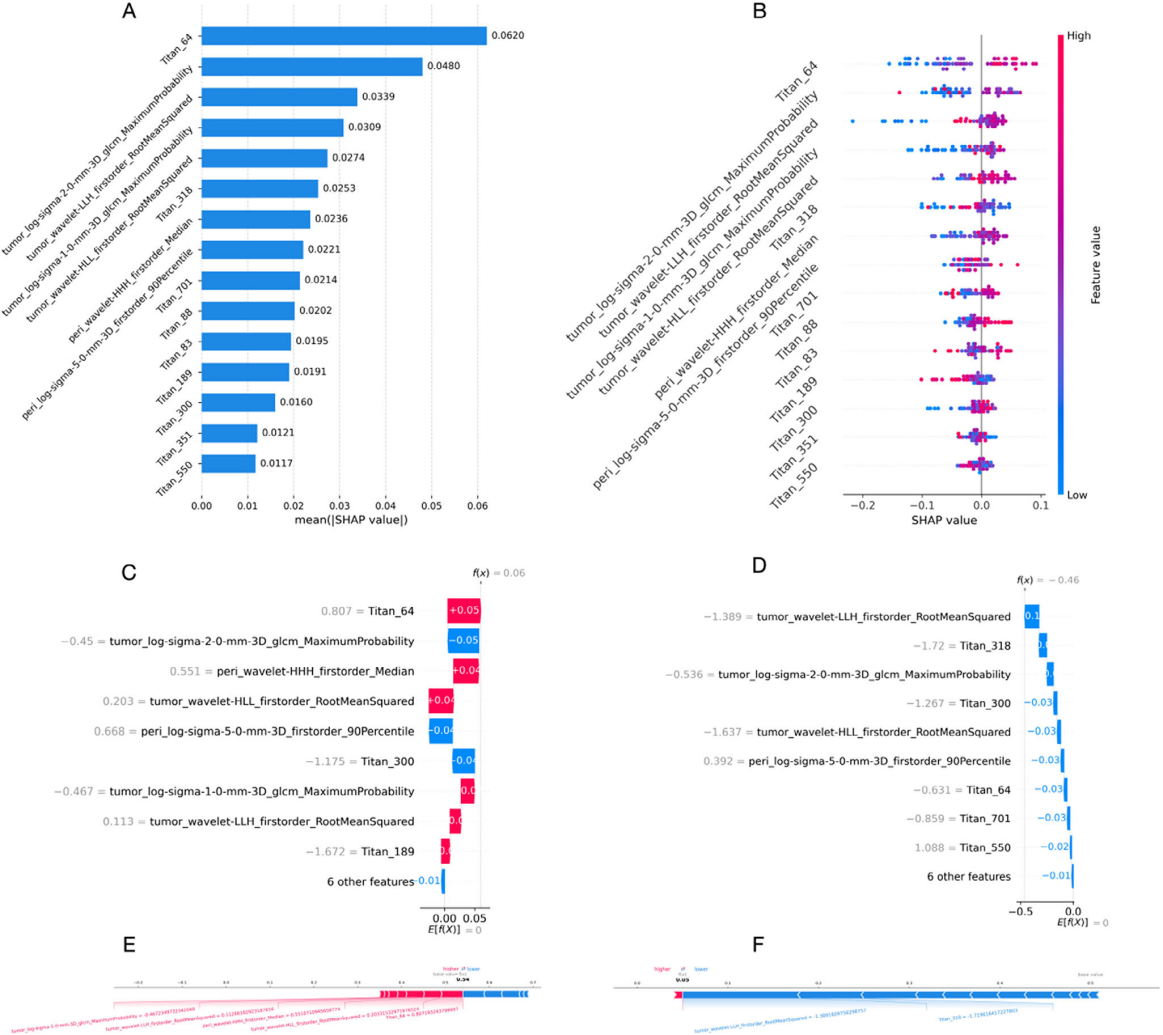
Explanation of the model predictions via the SHAP method. Cohort‐level Interpretation: (A) SHAP summary plot (mean |SHAP|) and (B) Beeswarm plot, showing the overall feature importance and effect distribution across the internal and external test set. Individual‐level Interpretation: (C,E) Waterfall and force plots detailing the feature contributions for two high‐risk ER patients. (D,F) Corresponding plots for two low‐risk ER patients. The base value (gray line) denotes the average prediction. The direction and magnitude of each arrow dictate the shift of f(x) from this average, classifying the instance as ER (red) or LR (blue). Abbreviations: SHAP: SHapley Additive exPlanations; ER: Early Recurrence; LR: Late Recurrence.

To interpret the contribution of each feature to the model's prediction of ER, SHAP analysis was performed on the RF model. The results presented in Figure 4 provide interpretations at both the cohort and individual levels. At the cohort level (Figure 4A,B), the analysis revealed the global importance of tumor‐region radiomic, peritumoral‐region radiomic, and pathological features. The top three most influential features were: the 64th pathological feature (mean |SHAP| = 0.0620), the tumor‐region radiomic feature log‐sigma‐2‐0‐mm‐3D_glcm_MaximumProbability (0.0480), and the tumor‐region radiomic feature mass_wavelet‐LLH_firstorder_RootMeanSquared (0.0399). The model's individual‐level utility is demonstrated in two cases: it correctly identified a patient who experienced ER assigning a 56.0% probability (above the 43.2% cut‐off value) (Figure 4C,E); conversely, it gave only a 4.0% probability to a patient who remained recurrence‐free (Figure 4D,F), accurately reflecting their distinct clinical outcomes.

### 2.3 Generation of the Single‐Modality Models

We constructed single‐modality models using the same feature selection and modeling pipeline detailed in Section 2.2, but applied separately to each individual feature type: tumor‐region radiomic features, peritumoral‐region radiomic features, and pathological features. Specifically, 16 tumor‐region radiomic features, 15 peritumoral‐region radiomic features, and 15 pathological features were selected via univariable analysis and LASSO regression. The feature selection process using LASSO regression for each feature type is shown in Figure S1 (tumor region), Figure S2 (peritumoral region), and Figure S3 (pathological features), respectively. For each feature set, 11 distinct machine learning models were developed, with the optimal model for each modality determined by its performance on the internal test set. The detailed results of all these models on each dataset are provided in Tables S5–S13. The final models selected were AB for the tumor radiomics model, DT for the peritumoral radiomics model, and SVM for the pathological model.

**FIGURE 5:**
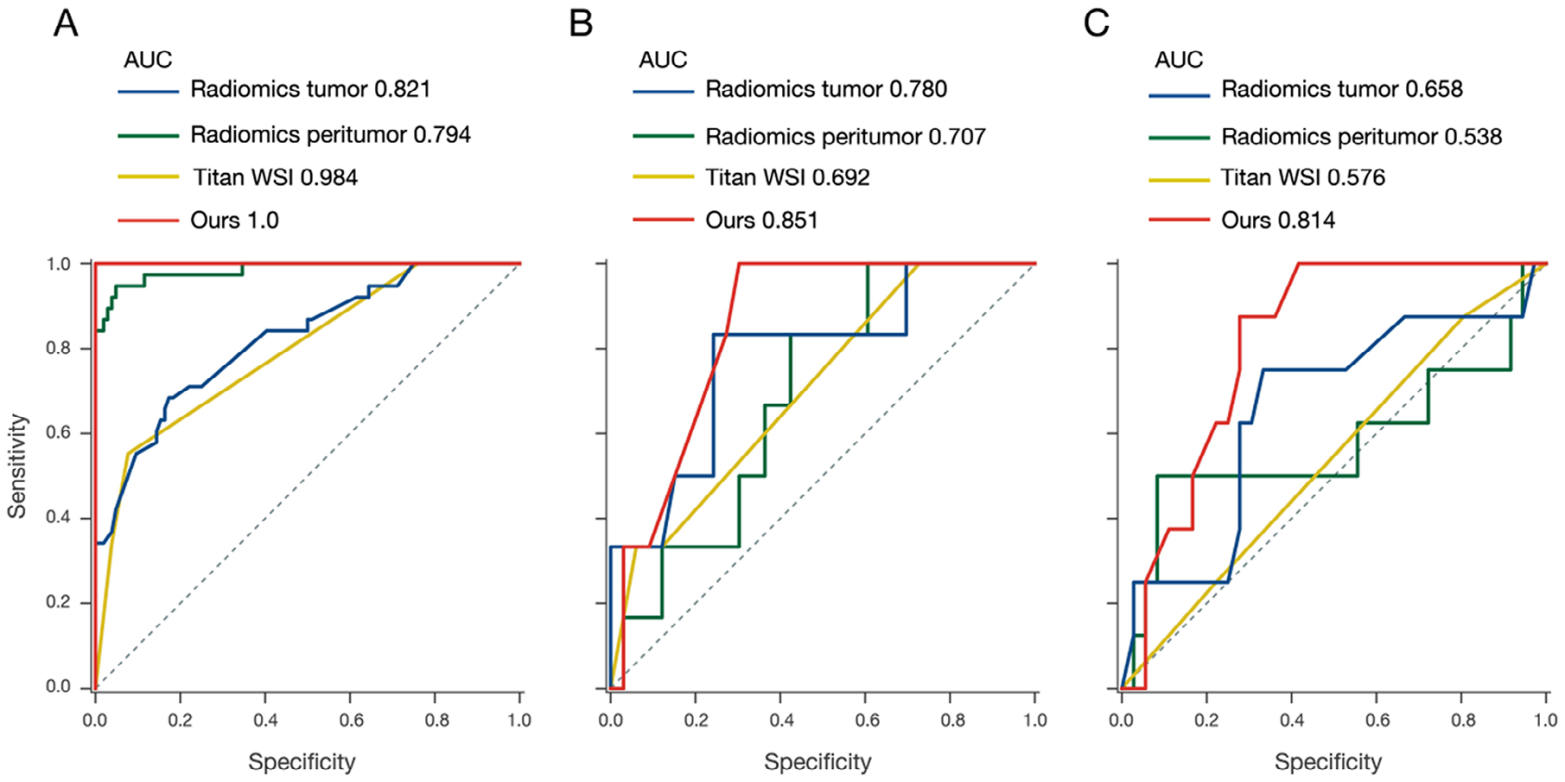
Performance comparison of the integrated Rad‐Path model vs. the single‐modality models across the training, internal test, and external test sets. ROC curves illustrate the predictive performance of the three single‐modality models (based on tumor‐region radiomics, peritumoral‐region radiomics, and pathological features) and the Rad‐Path model for ER patients in the (A) training set, (B) internal test set, and (C) external test set. Abbreviations: Rad‐Path: radiomics‐pathology; AUC: Area Under the Curve; WSI: Whole Slide Imaging; ROC: Receiver Operating Characteristic; ER: Early Recurrence.

The ROC curves for these optimal single‐modality models across the training, internal test, and external test sets are presented in Figure 5, with comprehensive performance metrics listed in Table S14. Analysis of the results indicated that the pathological model achieved the highest AUC on the training set (0.984), while the tumor radiomic model demonstrated the best performance on both the internal test set (AUC = 0.780) and the external test set (AUC = 0.658). Notably, the performance of all single‐modality models was consistently inferior to that of the integrated Rad‐Path model across all datasets (training set 1.0 vs. 0.984; internal test set: 0.851 vs. 0.780; external test set: 0.814 vs. 0.658).

The external performance of the single‐modality models revealed important considerations. Notably, the pathology‐only model exhibited the most significant performance drop from training (AUC 0.984) to external validation (AUC 0.576), indicating a high susceptibility to overfitting and poor generalizability across institutions. This likely reflects sensitivity to technical variations in slide preparation, staining, and scanning. In contrast, the tumor‐region radiomics model demonstrated more consistent, though moderate, external performance (AUC 0.658). The integration of modalities in the final Rad‐Path model yielded a substantial improvement (AUC 0.814), highlighting the complementary value of combining radiological and pathological data. Among the 15 features selected for the final model, 6 were radiomic, and 9 were pathological. SHAP analysis (Figure 4) confirmed that features from both modalities contributed to predictions, with the top‐ranking pathological feature demonstrating a mean |SHAP| value of 0.0620.

A critical evaluation of the integrated model's performance reveals a necessary trade‐off. In the internal and external test sets, the model achieved sensitivities of 0.500 and 0.625, respectively. This indicates that a substantial proportion of true ER patients were not classified as high‐risk. The corresponding specificities were 0.848 and 0.778, meaning that a smaller fraction of non‐ER patients were incorrectly flagged.

### 2.4 scRNA‐seq Analysis of Predicted ER and LR Groups

**FIGURE 6:**
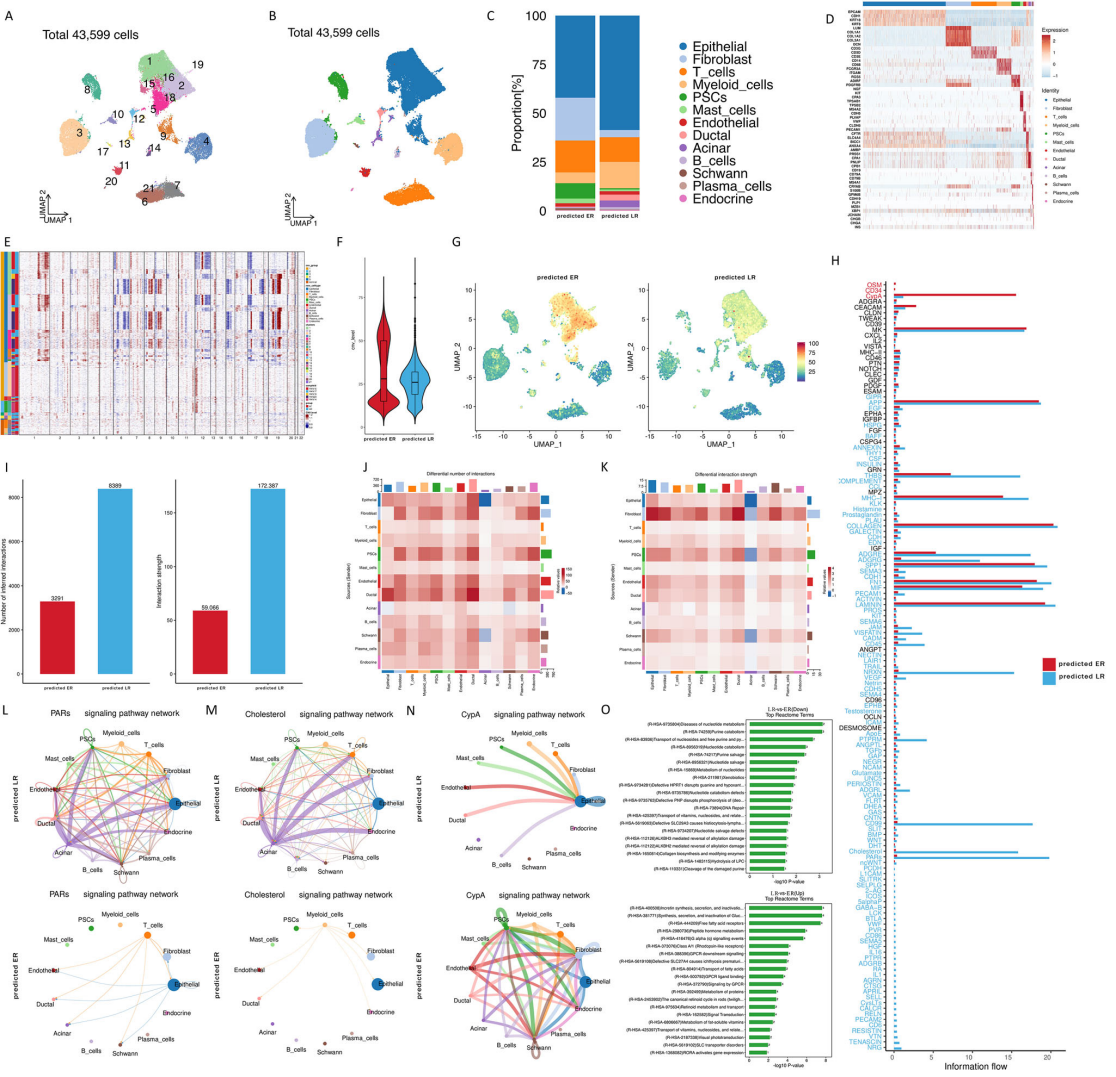
scRNA‐seq and cell–cell communication analysis comparing predicted ER and LR groups. (A) UMAP visualization of unsupervised clustering results from single‐cell transcriptomes, identifying 21 distinct clusters. (B) Annotation of the 17 major cell types identified. (C) Sample‐wise relative abundances of the annotated cell types. (D) Heatmap depicting marker gene expression across the 17 cell types. (E–G) Copy number variation (CNV) analyses: overall CNV landscape (E), group‐wise CNV distribution shown by violin plots (F), and UMAP visualization of inferred CNVs (G). (H) Bar plots summarizing the information flow of 138 L‐R pairs in each group. I) Intercellular interaction number and strength between predicted ER and LR groups. (J–K) Heatmaps depict the number (J) and strength (K) of cell–cell communications among cell types. Red and blue colors indicate interactions that are upregulated and downregulated, respectively, in the predicted LR group illustrating the number (J) and strength (K) of cell–cell communications across cell types (red cube indicated upregulated cell–cell communication in predicted LR group, blue cube indicated downregulated cell–cell communication in predicted LR group). The top and right bar plots indicate incoming and outgoing signaling, respectively. (L–N) Circle plots of the top two significantly downregulated (L,M) and upregulated (N) ligand‐receptor pairs in the ER group (the OSM pathway is excluded due to zero information flow in A0). (O) Reactome pathway enrichment analysis of differential metabolites identified from metabolomics between predicted ER and LR groups. Abbreviations: UMAP: uniform manifold approximation and projection; ER: Early Recurrence; LR: Late Recurrence; PSCs: pancreatic stellate cells; PARs: Protease‐Activated Receptors; CypA: Cyclophilin A; CNV: Copy number variation; OSM: Oncostatin M.

Five PDAC specimens were prospectively collected from three patients predicted to have ER and two LR based on our established Rad‐Path model. These samples were categorized into predicted ER and LR groups for downstream analysis. After quality control and preprocessing, 43 599 high‐quality cells were retained for dimensional reduction and clustering. Unsupervised clustering visualized by uniform manifold approximation and projection (UMAP) revealed 21 distinct cell clusters (Figure 6A). These clusters were annotated into 17 major cell types according to established marker genes (Figure 6B–D). Notably, fibroblasts and pancreatic stellate cells (PSCs) were substantially enriched in the predicted ER group compared to the LR group (Figure 6C). (CNV) analysis indicated elevated genomic instability in the ER group (Figure 6E,F), particularly within epithelial cells (Figure 6G). Kyoto Encyclopedia of Genes and Genomes (KEGG) pathway analysis further identified upregulation of protein synthesis‐related pathways—such as “ribosome” and “protein export”—in fibroblasts and PSCs from the ER group. In contrast, pathways involved in cell junction organization were predominantly downregulated (Figures S4 and S5).

### 2.5 Differential Cell–Cell Communication Between Predicted ER and LR Groups

To uncover cell–cell communication alterations underlying the divergence between predicted ER and LR groups, we identified 110 dysregulated ligand‐receptor (L‐R) pairs (Figure 6H). The ER group showed a marked reduction in both the number and strength of intercellular interactions, with the majority of differentially expressed L‐R pairs being downregulated (Figure 6I–K). Differences in interaction numbers were most apparent in the outgoing and incoming signals of epithelial cells, fibroblasts, stellate cells, and ductal cells, as well as in the incoming signals of endocrine cells (Figure 6J,K). Differences in interaction strength were mainly observed in the outgoing signals from fibroblasts and incoming signals to acinar cells. The two most downregulated L‐R pairs in the predicted ER group belonged to the Protease‐Activated Receptors (PARs) and Cholesterol pathways (Figure 6L,M). The PARs pathway comprised eight genes. Among these, F2R (encoding PAR1), expressed predominantly in fibroblasts, was notably upregulated in the ER group (Figure S6). Conversely, the two most upregulated L‐R pairs in the predicted ER group were linked to Cyclophilin A (CypA) (Figure 6N) and Oncostatin M (OSM) signaling. The OSM‐related pair was excluded from the visualization due to an absent signal in the LR group. Interestingly, the predicted ER group exhibited downregulated metabolites associated with RORA signaling (Figure S7), as identified by Reactome enrichment analysis of metabolomics data (Figure 6O). Concurrently, the analysis further revealed a significant upregulation of metabolites related to collagen biosynthesis and modifying enzymes in this group (Figure 6O).

### 2.6 Spatial Transcriptome

**FIGURE 7:**
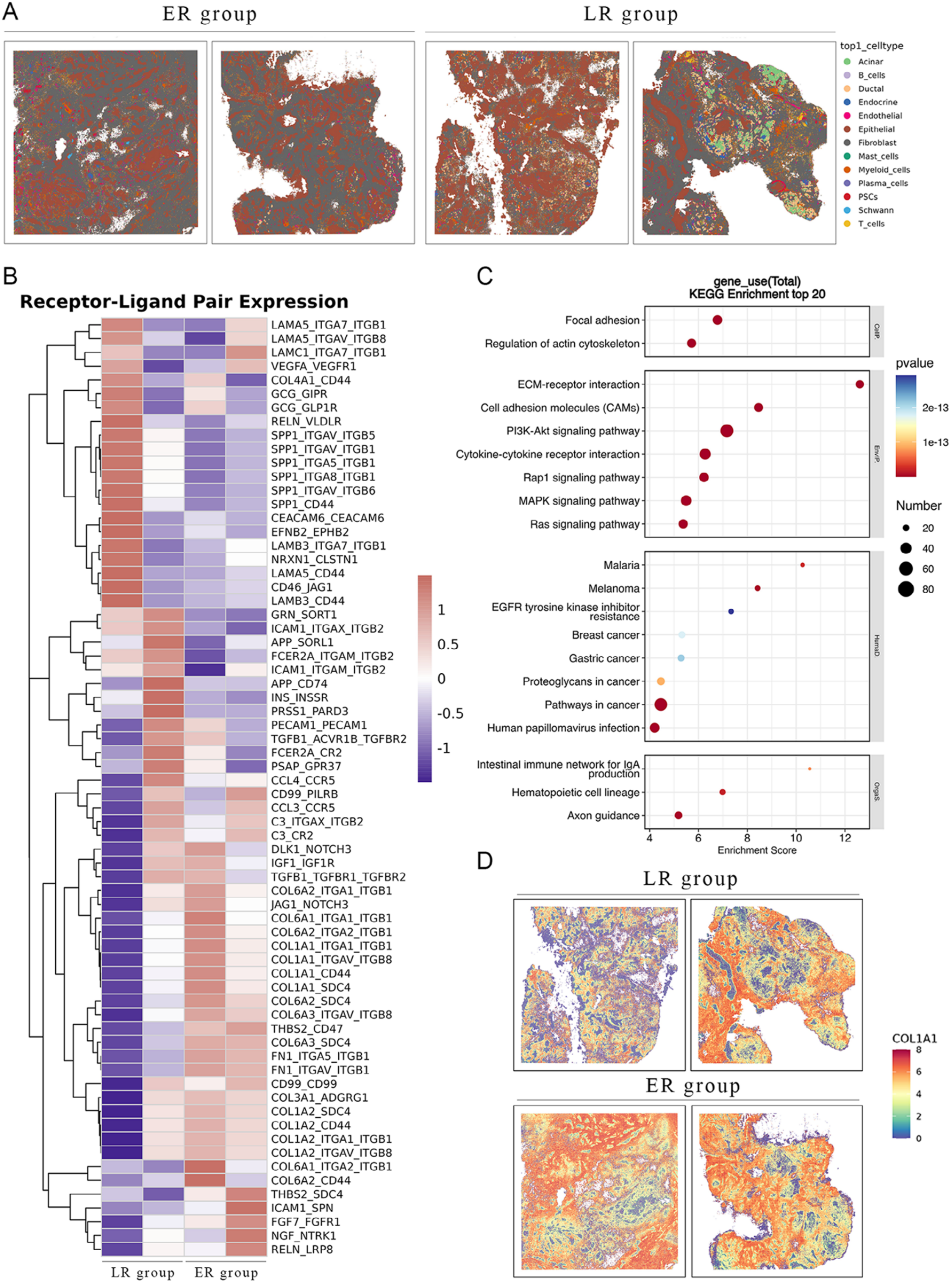
Spatial transcriptome analysis comparing predicted ER and LR groups. (A) Annotation of the most abundant cell type for each spot. (B) Heatmap displaying the expression of 67 L‐R pairs across predicted ER and LR patient samples. (C) KEGG functional enrichment analysis of the detected L‐R genes. (D) Representative spatial expression pattern of the ligand COL1A1 in predicted LR and ER groups. Abbreviations: ER: Early Recurrence; LR: Late Recurrence; PSCs: pancreatic stellate cells; ECM: Extracellular Matrix; KEGG: Kyoto Encyclopedia of Genes and Genomes.

To validate and expand on the scRNA‐seq findings, we performed ST sequencing on four prospectively collected tissue sections (two each from predicted ER and LR groups) using the 10 × Visium HD platform. One of these patients also underwent scRNA‐seq. After quality control, each section yielded between 487 282 and 666 848 high‐quality barcoded spots, with an average unique molecular identifier (UMI) count per spot ranging from 458 to 539. We then applied robust cell type decomposition (RCTD) to infer cell‐type composition for each spot based on our scRNA‐seq reference. The predominant cell type in each spot is visualized in Figure 7A. Consistent with the scRNA‐seq data, the ER group exhibited a higher proportion of fibroblasts compared to the LR group. A total of 67 L‐R pairs were identified through cell–cell communication analysis (Figure 7B). KEGG enrichment analysis of the corresponding genes revealed a strong association with cell adhesion and extracellular matrix (ECM) interactions (Figure 7C). For example, COL1A1 (Figure 7D)—a ligand gene upregulated in the predicted ER group—encodes the main component of the most abundant collagen in the ECM [29].

### 2.7 Association of subTME Phenotypes With Model‐Predicted Recurrence Risk

**FIGURE 8:**
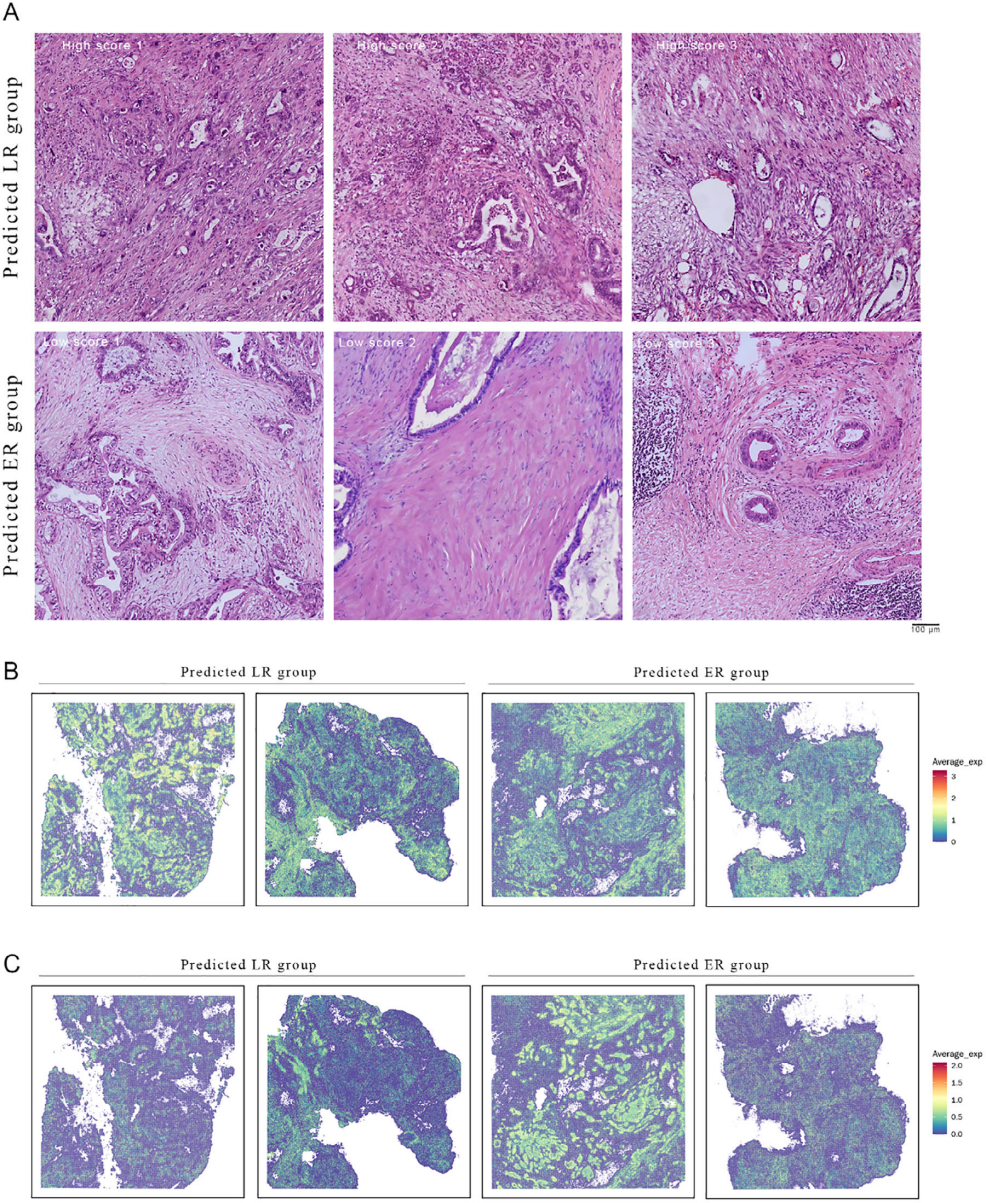
Pathological and spatial transcriptomic features of subTME phenotypes in predicted ER vs. LR groups. (A) Compared to the LR group (bottom), the predicted ER group (top) is characterized by a greater abundance of active fibroblasts, thereby illustrating a “reactive‐dominant” subTME in ER vs. a “deserted‐dominant” subTME in LR. (B,C) Spatial transcriptomics validates the corresponding molecular profiles, showing higher expression of (B) “deserted” phenotype markers in the LR group and (C) “reactive” phenotype markers in the ER group. Abbreviations: ER: Early Recurrence; LR: Late Recurrence; subTME: Subtumor Microenvironment.

To validate the findings from our scRNA‐seq and ST analyses, we histologically evaluated the ECM in tissue sections from the top three patients with high (predicted ER) and low (predicted LR) model scores, respectively, in the internal validation cohort. The evaluation was performed in a blinded manner by an experienced pathologist. The observed ECM characteristics closely matched a previously established subtumor microenvironment (subTME) phenotypic framework, which was originally defined by Grünwald et al. based on distinct pathologic features [30]. The “reactive‐dominant” subTME, enriched with active fibroblasts relative to the “deserted‐dominant” subtype, demonstrated a strong correlation with aggressive tumor behaviors—including migration and proliferation—and shorter DFS post‐resection [30, 31, 32]. Specifically, tissue sections from the predicted ER group were classified as “reactive‐dominant,” in contrast to the “deserted‐dominant” phenotype found in the LR group (Figure 8A). The evaluation results of the six patients are summarized in Table S15. ST imaging further confirmed that the expression patterns of the reported marker genes for “reactive” (e.g., FAP, ACAT2, S100A4, NNMT, IL6, IL1B) and “deserted” (e.g., ENO1, ITGB1, CD44, LGALS1, KIT) subTMEs [30] were consistent with the predictions of the Rad‐Path model (Figure 8B,C).

In summary, our multi‐omics investigation—spanning scRNA‐seq, ST, and histopathological validation—consistently demonstrates that ECM remodeling [33] is a central biological determinant underlying the Rad‐Path model's ability to stratify ER vs. LR in PDAC.

## 3 Discussion

In this retrospective‐prospective study, we developed and validated an integrated model combining contrast‐enhanced CT (CECT)‐based radiomics and computational pathology to predict ER of PDAC following radical resection. The optimal model demonstrated consistent predictive performance across training, internal validation, and external validation cohorts. To elucidate the decision logic and biological basis of the model, we performed a multi‐faceted interpretability analysis. Using SHAP, we quantified the contribution of each feature to model predictions, enabling both cohort‐level interpretation—by ranking global feature importance—and individual‐level analysis—by decomposing each prediction into feature‐specific contributions, thereby supporting personalized clinical reasoning and outlier identification. Complementarily, we integrated scRNA‐seq with ST to uncover key cellular and spatial molecular processes associated with model predictions, thereby enhancing biological interpretability at the single‐cell and tissue microenvironment levels.

This study developed and validated a series of models for predicting 6‐month PDAC recurrence. The integrated Rad‐Path model demonstrated robust performance, achieving AUCs of 0.851 and 0.814 on the internal and external test sets, respectively. Notably, it consistently outperformed all single‐modality models across training, internal, and external validation cohorts. This consistent advantage underscores the synergistic effect of combining radiomic and pathological features, suggesting that the integrated approach enables more holistic tumor profiling than any single data modality alone. The Rad‐Path model demonstrated strong discriminative power even in cases with highly similar imaging appearances (Figure S8). The ability of the model to correctly classify ER and LR cases that are visually indistinguishable on CT highlights its capacity to extract critical biological information beyond macro‐morphology. This evidence supports the potential of the Rad‐Path model as a complementary tool to traditional imaging, enhancing the precision of post‐operative prognosis and clinical management.

Our findings underscore both the promise and practical challenges of multimodal integration. The pathology‐only model demonstrated marked performance decline in external validation, a recognized issue attributed to technical heterogeneity in histopathology workflows. However, its integration with radiomics substantially improved overall model robustness and accuracy. This pattern suggests that radiomic features offer a relatively stable, imaging‐platform‐independent foundation, whereas computational pathology features contribute complementary biological information not accessible through radiology alone. The wide confidence interval for the external AUC of the pathology model further highlights the need for larger, multi‐center cohorts to obtain more reliable estimates. Future efforts should prioritize the development and application of advanced standardization and domain adaptation techniques to improve the portability of pathology‐based features across institutions.

We acknowledge that the perfect AUC (1.000) achieved on the training set by some models (i.e., GB, RF, XGB in Table 2) is a clear indicator of overfitting. This underscores the critical importance of rigorous external validation. In selecting our final model, we deliberately prioritized consistent performance on the independent validation cohorts over the training metrics. The Random Forest model, despite its perfect training score, demonstrated the most stable and generalizable performance, maintaining high AUCs on both the internal (0.851) and external (0.814) test sets. This relative resistance to overfitting is likely because of its ensemble architecture. The observed performance gap between cohorts highlights a challenge for model generalizability, attributable to real‐world clinical and technical variability. Nevertheless, the maintained efficacy in the external setting supports the validity of our integrated approach.

The substantial differences in classifier performance require further discussion. Linear models such as LDA and LR consistently underperformed across all datasets, with internal validation AUCs as low as 0.455–0.465. This is unsurprising given the high‐dimensional, non‐linear structure of the integrated radiomic and pathological feature space; linear decision boundaries are inherently limited in their capacity to separate such complex representations [34, 35]. In contrast, ensemble tree‐based methods, including RF, GB, and XGB, consistently achieved the highest predictive accuracy. This advantage is well documented and reflects their ability to model non‐linear feature interactions, accommodate mixed data types, and remain robust to irrelevant predictors [36, 37].

Although GB yielded a marginally higher external AUC (0.819 vs. 0.814), we selected RF as the final model based on its more stable performance across both validation cohorts and its smaller performance drop from training to validation. The selection of Random Forest was based on its superior stability and generalizability, both critical for clinical deployment [38, 39]. Notably, even among ensemble methods, overfitting was evident in models such as XGBoost and SVM, reinforcing the necessity of rigorous external validation and the value of simpler, well‐regularized architectures when sample sizes are constrained.

Existing models for PDAC ER prediction, despite their predictive value [12, 24, 40], share a critical limitation: dependence on a single data modality provides a narrow window into the underlying tumor biology. The integration of pathological features leads to more comprehensive models and superior predictive performance. For instance, combining MRI with pathological imaging has proven highly effective in predicting pathological complete response to neoadjuvant chemotherapy in breast cancer [41] and pathological response to neoadjuvant chemoradiotherapy in rectal cancer [42]. Unfortunately, a key limitation persists in most integrated imaging‐pathology models: their predictive power is still fundamentally constrained by limited biological interpretability, often resulting in an opaque decision‐making process that impedes clinical adoption [11]. Innovatively, our model distinguishes itself by not only integrating rich biological data but also through the explicit inclusion of scRNA‐seq and ST to uncover the biological drivers of its performance.

Although our model was primarily designed to predict ER in PDAC patients, preliminary findings from scRNA‐seq, ST, and histopathology converge to suggest a central role of the ECM—primarily produced by fibroblasts and PSCs [43]. In predicted ER patients, scRNA‐seq revealed an elevated abundance of fibroblasts and PSCs coupled with reduced intercellular communication. Notably, the two most dysregulated pathways in cell communication—OSM and cholesterol signaling—have previously been established as mediators of pancreatic fibrosis [44, 45]. The OSM has been identified as an inducer of LOXL2 expression, which promotes tumor progression and metastasis [46]. Moreover, OSM is also known to reprogram cancer‐associated fibroblasts into a tumor‐promoting phenotype in pancreatic and breast cancer models [44, 47]. Within the Cholesterol pathway, RAR‐Related Orphan Receptor A (RORA)—a nuclear receptor serving as its core regulator—has been reported to suppress tumor growth and metastasis and protect against pancreatic fibrosis [45, 48, 49]. The other dysregulated genes, including PAR1 and CypA, are also established promoters of cancer progression. PAR1 activation promotes tumor growth and metastasis via STAT3 and NF‐κB signaling in multiple cancer types [50, 51]. Recent studies have demonstrated that CypA is closely associated with the proliferation, invasion, metastasis, and recurrence of gastric cancer [30, 31, 32]. Collectively, our findings indicated that enhanced PAR1, CypA, and OSM signaling, together with suppressed Cholesterol pathway activity, may drive the formation of a pro‐aggressive tumor microenvironment that favors ER.

ST analysis confirmed the expansion of fibroblasts in predicted ER cases, aligning with scRNA‐seq findings. Functional enrichment further highlighted enhanced ECM‐related pathways in these patients. For instance, COL1A1, which encodes the most abundant collagen in the ECM and was upregulated in ER patients, has been associated with metastasis, tumor growth, and immune infiltration across various cancers [29]. Histopathological and ST evaluation corroborated differences in ECM composition, revealing that ER patients predominantly presented a “reactive‐dominant” subTME characterized by abundant activated fibroblasts—a pattern associated with enhanced tumor aggressiveness and invasion [30]. Moreover, findings from our prior spatial metabolomics study involving six additional pancreatic cancer sections [52], align with the current results: L‐R pairs upregulated in hypermetabolic regions partially overlapped with those upregulated in the predicted ER group, such as COL1A1‐CD44, COL1A2‐(ITGAV+ITGB8), SPP1‐CD44, THBS2‐SDC4, and COL1A1‐SDC4.

Previous studies have proposed that ER following surgical resection may originate from pre‐existing occult micrometastases [53, 54]. Further evidence indicates that ER may be facilitated by mechanical stimulation during surgery and partially driven by postoperative immunosuppression [54, 55]. This view is supported by mathematical modeling, which estimates a growth period of about 21.9 months for metastatic cells to form a 1‐cm^3^ mass [5, 56]. We therefore propose a mechanism whereby the model predicts ER by identifying features in the primary tumor that reflect an inherent potential for micrometastasis. Given that dissemination initiates metastasis and our model was trained specifically on primary tumor features most relevant to this stage. It is significant, then, that its predictions centered on epithelial‐to‐mesenchymal transition and ECM remodeling—two established drivers of dissemination [33, 57, 58]. Building on the known role of fibroblasts and PSCs in ECM remodeling—both of which were enriched in the predicted ER group—our findings suggest that ECM remodeling serves as a key biological basis for the differential outcomes identified by our model.

Prior studies have established radiomic models for predicting fibroblast activation protein expression in PDAC [59, 60], and Xie et al. linked a noninvasive intratumoral heterogeneity classification model in hepatocellular carcinoma to fibroblast enrichment and ECM remodeling [61]. In contrast, our work provides the first evidence that an ER prediction model in R0‐resected PDAC is fundamentally associated with tumor ECM characteristics. This finding underscores the need to further investigate the roles of fibroblast activation and ECM remodeling in PDAC recurrence, and suggests that microenvironmental features may also enhance models predicting drug response or overall survival.

Our study has several limitations. First, as the predictive model was developed using retrospectively collected data from resected PDAC patients, it may be subject to selection bias, necessitating prospective validation to confirm its accuracy and generalizability. Second, the lack of standardized algorithms for radiomic feature processing across studies underscores the need for methodological harmonization in the field. Third, while integrated scRNA‐seq and ST analyses offered mechanistically plausible insights supported by pathological correlation, further functional experiments are required to establish causal relationships. Fourth, the moderate sensitivity of our current model warrants careful consideration for its clinical role. A sensitivity of 50%–60% implies that many patients destined for early recurrence would not be pre‐emptively identified. Therefore, the model in its present form is not suitable as a standalone rule for withholding adjuvant therapy, where missing a high‐risk case carries significant consequences. Instead, its primary value may be as a preoperative risk stratification and decision‐support tool. It could robustly identify a subgroup with a very high predicted probability of ER, who might be prioritized for neoadjuvant therapy, more intensive surveillance, or inclusion in clinical trials. Conversely, a low predicted risk could reinforce the rationale for standard management. The optimal sensitivity threshold is context‐dependent and should be calibrated based on the clinical cost of a false‐negative vs. a false‐positive result in future implementation studies. Fifth, our study is limited by the overfitting observed during model development. We acknowledge that the perfect training performance (AUC = 1.000) of certain models indicates overfitting. Therefore, we prioritized generalization and selected the final model based on its consistent results across independent validation cohorts. Future work employing larger prospective cohorts is warranted to further improve model robustness. Sixth, the relatively small sample size of the prospective cohort may limit its statistical power, although the findings consistently align with a central role for the ECM.

## 4 Conclusions

In conclusion, we have not only developed and validated a multimodal predictive model but also elucidated its mechanistic basis via scRNA‐seq and ST, identifying ECM remodeling as the foundational biological process. This interpretable approach holds translational potential to support personalized postoperative management and inspires future investigation into microenvironment‐driven recurrence.

## 5 Materials and Methods

Ethical approval was obtained from the institutional review board for this retrospective‐prospective study (I‐25PJ1752). Informed consent was waived for retrospective data use, whereas written consent was obtained from all prospective cohort patients for scRNA‐seq, ST, and pathological studies.

### 5.1 Patient Cohorts

This study enrolled patients with pathologically confirmed PDAC who underwent surgical resection at two independent institutions, retrospectively. The primary cohort (Institution 1, July 2018–June 2023) was used for model development and internal validation, while an independent external validation cohort was obtained from Institution 2 (January 2022–June 2023). There was no patient overlap between the two institutions.

Inclusion required: (1) histopathologically verified PDAC with available slides; (2) R0 resection margin status; (3) CECT within 2 weeks prior to surgery. Key exclusion criteria comprised: (1) incomplete clinical, imaging, or pathological data; (2) preoperative biliopancreatic stent placement; (3) other tumor history; (4) loss to follow‐up; or (5) receipt of neoadjuvant therapy.

Collected clinical variables included demographics, clinical history, preoperative laboratory parameters (blood tests, liver and renal function tests, and serum tumor markers including carcinoembryonic antigen, carbohydrate antigen 19‐9, and cancer antigen 125, all measured within one week before surgery), pathological characteristics, and RFS. The CT scanning protocols in the two institutions were summarized in Tables S16 and S17.

From December 2024 to April 2025, we prospectively enrolled patients with PDAC undergoing surgical resection at Institution 1. For each patient, we collected preoperative CECT scans, histopathological images, laboratory results, clinical data, and resected tumor specimens. A detailed patient recruitment flowchart is presented in Figure 1.

The class distribution in the training set was 38 patients with ER and 104 patients with LR (ER:LR ratio ≈ 1:3). To account for potential class imbalance during model evaluation, in addition to standard metrics, balanced accuracy and F1‐score were also calculated and are reported in Tables S2–S13.

### 5.2 Radiomic Feature Extraction

Image segmentation was performed by a senior abdominal radiologist with 15 years of experience, using 3D Slicer (v5.6.2). The radiologist manually delineated the regions of PDAC on each slice of the pancreatic parenchymal phase images of CECT, remaining blinded to all clinical and pathological information. The segmentations were then reviewed by two additional radiologists, and any discrepancies were resolved through consensus. Following the manual segmentation, a 3 mm peritumoral margin was automatically generated using the BinaryDilate function from the SimpleITK library (v2.5.2).

Radiomic features were extracted from both the PDAC and the surrounding peritumoral areas using the PyRadiomics package (v3.1.0). A total of 1218 features were calculated for each region, including six categories: 14 shape descriptors, 252 first‐order statistics, 308 gray‐level co‐occurrence matrix features, 196 gray‐level dependence matrix features, 224 gray‐level run‐length matrix features, and 224 gray‐level size zone matrix features. In total, 2436 features were obtained per patient for further modeling.

### 5.3 Whole‐Slide Imaging

The Hematoxylin and Eosin (H&E)‐stained slides for whole‐slide imaging were sourced from collaborating pathology departments, encompassing both retrospective and prospective cohorts. For each case, the slide exhibiting the most substantial tumor component was selected by an experienced pathologist (8 years). The selected slides were scanned using a whole‐slide scanner (3DHistech Pannoramic 250 or KFBIO KF‐PRO‐400‐HI).

### 5.4 Pathology Slide Feature Extraction

Whole‐slide images (WSIs) were processed using the TITAN model to extract slide‐level feature representations [62]. To address potential technical variability in WSIs arising from different scanners and staining protocols, all WSIs underwent a standard preprocessing pipeline. Each WSI was first preprocessed to identify tissue areas, from which non‐overlapping 512 × 512 pixel patches were extracted at 20 × magnification. These patches were encoded into 768‐dimensional feature vectors using the pretrained CONCHv1.5 model. The resulting features were arranged into a 2D grid that mirrored the spatial layout of the tissue. This grid was input into TITAN's slide encoder, a Vision Transformer model that employed 2D Attention with Linear Biases (ALiBi) for positional encoding. ALiBi allowed the model to generalize across various resolutions without retraining. Long‐range dependencies across patches were modeled through self‐attention mechanisms. Finally, attention pooling was applied to derive a slide‐level feature embedding. A trainable query token aggregated contextual information from all patches, yielding a 768‐dimensional feature vector, which was used for further modeling.

### 5.5 Feature Selection

The combined 3204 radiomic and pathological features underwent a multi‐step feature selection process. First, Z‐score normalization was applied to standardize the features for comparability. Second, univariate selection was performed, where statistically significant features were identified using hypothesis testing. Finally, redundant features were reduced using LASSO regression with 5‐fold cross‐validation, which helped improve interpretability and mitigate multicollinearity. These processes were implemented in Python 3.9 using the Scikit‐learn library (v1.7.2).

### 5.6 Model Development

Eleven standard machine learning classifiers were employed to construct predictive models using the selected features. The model development process involved three key steps: dataset splitting, model training, and model validation. Initially, data from Institution 1 were randomly divided into a training set and an internal test set, while data from Institution 2 served as the external test set. For model training, Scikit‐learn (v1.7.2) was used, with the input features consisting of the selected radiomic and pathological slide features. Hyperparameter optimization was carried out using GridSearchCV, which enabled the identification of optimal hyperparameters through cross‐validation, thus enhancing the model's performance and robustness. Finally, the performance of the model was evaluated using both the internal and external test sets to assess its generalizability and reliability.

### 5.7 Single‐Cell RNA Sequencing

#### 5.7.1 Single‐Cell Suspension Preparation and Library Construction

Under sterile conditions, fresh tissues were washed with cold Dulbecco's phosphate‐buffered saline, minced, and digested in RPMI 1640 (Corning, cat. no. 10‐040‐CVR), 0.04% BSA (MACS, cat. no. 1000076), and 0.2% collagenase II (Gibco, cat. no. 17101015) solution at 37°C for 30–60 min. The cell suspension was filtered through a 40 µm strainer, centrifuged, and treated with red blood cell lysis buffer. Cell viability and concentration were determined using an automated cell counter with Trypan Blue staining. The prepared suspension was diluted to achieve a density of 700–1200 cells/µL. Single‐cell libraries were constructed with the 10× Genomics Chromium Next GEM Single Cell 3′ Kit v3.1 and sequenced on an Illumina Nova 6000 platform.

#### 5.7.2 scRNA‐seq Data Analysis

Raw sequencing data were processed using Cell Ranger (v9.0.1) for alignment and UMI counting with the GRCh38 human reference genome. Downstream analysis was performed in Seurat (v4.0.0). Quality control excluded cells with <200 genes, <1000 UMIs, >10% mitochondrial genes, or >5% hemoglobin genes. Doublets were removed using DoubletFinder (v2.0.3) [63]. Gene expression values were normalized by the “LogNormalize” method, and the top 2000 highly variable genes were selected for further analysis. Dimensionality reduction was performed using the RunPCA function. Clustering was conducted with the FindClusters function and visualized with the RunUMAP function. Bonferroni correction was applied for multiple testing adjustments. Marker genes for each cluster were identified with the FindAllMarkers function, applying thresholds of |log_2_FC| > 0.58 and adjusted *p* < 0.05. Cell types were manually annotated by referring to established marker genes from published literature [64]. Functional enrichment analysis was performed via KEGG on R (version 4.0.3). For CNV estimation, the inferCNV package (v1.0.4) was applied. Gene expression values from scRNA‐seq data were analyzed per cell, using a cutoff of 0.1. Genes were ordered according to genomic location, and a moving‐average approach (window size = 101 genes) was employed to smooth expression values. These values were then mean‐centered. Epithelial cells were designated as malignant, while all other cells served as the normal reference. Denoising procedures were applied to produce the final CNV profiles. CellChat (v2.1.2) was employed to explore intercellular communication networks. A CellChat object was generated from a normalized gene expression matrix via the createCellChat function. Preprocessing, including identifyOverExpressedGenes, identifyOverExpressedInteractions, and projectData functions, were performed using default settings. Potential L‐R interactions were inferred by running computeCommunProb, filterCommunication (min.cells = 10), and computeCommunProbPathway. Finally, the aggregateNet function was used to summarize the overall cell–cell communication network.

### 5.8 Metabolomic Profiling

Freshly obtained pancreatic specimens were trimmed, surface‐dried, and embedded in optimal cutting temperature medium (SAKURA, 4583) before snap‐freezing on dry ice. All samples were maintained at −80°C until processing. Cryosectioning was performed on a Leica CM1950 cryostat at −20°C to generate 10 µm sections.

Tissue sections were subjected to metabolomic analysis using an AFADESI‐MSI system (Beijing Victor Technology Co., Ltd.) coupled to a Q Exactive hybrid quadrupole‐Orbitrap mass spectrometer (Thermo Scientific). The spray solvent consisted of acetonitrile/water (8:2, v/v) for negative ion mode, and acetonitrile/water (8:2, v/v) containing 0.1% formic acid for positive ion mode, delivered at 5 µL/min. Additional parameters included a nebulizing gas flow of 45 L/min, spray voltage of 7 kV, and distances of 3 mm from the sprayer to both the sample surface and the ion transfer tube. Mass spectrometry settings were as follows: resolution 70 000, scan range m/z 70–1000, AGC target 2 × 10^6^, maximum injection time 200 ms, S‐lens RF level 55, and capillary temperature 350°C.

Metabolite identification was performed by matching accurate mass and MS/MS spectra against HMDB, METLIN, and an in‐house SmetDB database with a 5‐ppm mass tolerance. Multivariate statistical analysis included principal component analysis (PCA) and orthogonal partial least squares‐discriminant analysis, with metabolites having variable importance in projection > 1.0 and *p* < 0.05 considered differentially abundant (Bonferroni correction). Reactome pathway enrichment was performed in R using clusterProfiler.

### 5.9 Spatial Transcriptomics

#### 5.9.1 Library Preparation and Sequencing

The sections for ST were mounted onto Visium slides (Sigma–Aldrich, P0425). Subsequent methanol fixation, H&E staining, brightfield imaging, and destaining steps followed the manufacturer's protocol (10x Genomics, CG000763). Probe hybridization, cDNA synthesis, and library construction were conducted using the Visium HD Gene Expression Reagent kit (10x Genomics, PN‐1000675) according to the user manual (CG000685). The BGI DNBSEQ‐T7 platform was used to sequence libraries with PE100 mode.

#### 5.9.2 Spatial Data Analysis

Raw sequencing data were aligned to the GRCh38 human genome and barcode‐processed using Space Ranger (v3.1.2). Tissue‐covered bins (8 × 8 µm) were selected for downstream analysis. The gene‐barcode matrix was imported into Seurat (v5.1.0) [65] and normalized via the “LogNormalize” function. We identified 3000 highly variable genes using the FindVariableFeatures function. Dimensionality reduction was performed using PCA, followed by graph‐based clustering with the FindClusters function. 2D visualization was achieved via UMAP. We employed spacexr [66] (v2.2.0) to estimate the cellular composition within each bin. The analysis was performed using the create. RCTD function with its standard settings, while requiring a minimum of more than one cell for every cell type and setting the threshold for UMI counts per pixel to exceed one. Additionally, the run. RCTD function was executed in “doublet” mode. Differentially expressed genes between clusters were identified using the function FindMarkers (test.use = presto), with significance thresholds set at |log2FC| > 0.58 and adjusted *p*‐value < 0.05 (Bonferroni correction). CellChat in R (v2.1.2) was employed to infer cell–cell communication. After importing the normalized expression matrix, ST data were supplemented with spatial coordinates (from the GetTissueCoordinates function in Seurat v4.3.0) and scale factors. The createCellChat function initialized the analysis object. Data preprocessing—including identifyOverExpressedGenes, identifyOverExpressedInteractions, and projectData functions—was performed under default parameters. Probable L‐R interactions were then derived by running computeCommunProb, filterCommunication (min.cells = 10), and computing pathway‐level communication. The aggregateNet function consolidated the network, and spatial signaling maps were generated by netVisual_aggregate to offer an intuitive visualization of the results. Functional enrichment analysis for KEGG pathways was carried out using the hypergeometric test in R (v4.0.3).

### 5.10 Histopathological Evaluation of subTME

Evaluation of subTME phenotypes was performed by an experienced pathologist (Dr. Wu, with 20 years of pathology experience) in a blinded manner. The phenotypes were classified as “deserted,” “intermediate,” or “reactive” based on previously established criteria [30]. The “deserted” subTEM was defined as an area containing sparse, thin fibroblasts embedded within fully matured collagen fibers and loose ECM. In contrast, the “reactive” subTEM was characterized by regions rich in plump fibroblasts with enlarged nuclei, situated in sparse ECM infiltrated with inflammatory cells. The region with intermediate features above was defined as “intermediate.”

### 5.11 Statistical Analysis

Continuous variables were presented as mean ± standard deviation or median (interquartile ranges) and compared using one‐way ANOVA or Kruskal–Wallis H test, as appropriate. Categorical variables were summarized as counts and percentages and compared using Pearson's chi‐square or Fisher's exact test. All statistical analyses were conducted using IBM SPSS Statistics (version 27.0.1). A two‐sided *p*‐value < 0.05 was considered statistically significant.

## Author Contributions

S.C. designed the study, organized the project, provided funding, and revised the manuscript; F.C. developed the main code and wrote/revised the manuscript; S.Z. collected data and multi‐omics samples, performed omics data analysis, and wrote/revised the manuscript; R.L. assisted in data collection, multi‐omics sample processing, and data analysis; W.Z. provided external validation cases and performed image segmentation; X.K. collected and analyzed pathological sections; J.W. prepared figures and visualization; Z.Z. and K.Z. performed image segmentation and patient follow‐up; D.D. and R.Z. extracted pathological features; Z.J. coordinated team management and provided guidance; M.S. provided expert advice; Z.W. assisted in case acquisition, coordinated collaborations, revised the manuscript, and provided funding; H.W. obtained and analyzed pathological sections and revised the manuscript; X.H. provided surgical specimens, prospectively collected patients, and revised the manuscript; N.H. provided external validation resources, funding, and supervision; H.X. provided funding, revised the manuscript, and coordinated collaborations.

## Funding

This study was funded by Beijing Natural Science Foundation (7232116, 7244524, L242061 and L252174), National Natural Science Foundation of China (82202268, 22232006, 82502418, and 82471950), National Key Research and Development Program of China (2023YFA0915304), CAMS Innovation Fund for Medical Sciences (CIFMS) (Grant No. 2024‐I2M‐ZD‐001), Peking Union Medical College Hospital Talent Cultivation Program (Category D) No.UHB11857, and National High‐Level Hospital Clinical Research Funding (2025‐PUMCH‐D‐002), Peking University Clinical Scientist Training Program, supported by “the Fundamental Research Funds for the Central Universities” (BMU2025PYJH043).

## Ethics Statement

Ethical approval was obtained from the institutional review boards of both participating medical centers for this retrospective‐prospective study. Informed consent was waived for retrospective data use, whereas written consent was obtained from all prospective cohort patients for scRNA‐seq, ST, and pathological studies.

## Consent

Informed consent for publication was obtained using the consent form provided by our institution. A copy of the signed form can be provided to the journal upon request.

## Conflicts of Interest

The authors declare no conflicts of interest.

## Supporting information




**Supporting File**: advs74947‐sup‐0001‐SuppMat.docx.

## Data Availability

The data that support the findings of this study are available from the corresponding author upon reasonable request.
