# Supplementary material for: Integrating Radiomics and Computational Pathology to Predict Early Recurrence of Pancreatic Ductal Adenocarcinoma and Uncover Its Biological Basis in Tumor Microenvironment
Source: Adv Sci (Weinh). 2026 Apr 13;13(32):e23985. doi: 10.1002/advs.202523985 (PMC13252617; doi:10.1002/advs.202523985)
Supplement: Supplementary file 1 — Supporting File: advs74947‐sup‐0001‐SuppMat.docx. [file ADVS-13-e23985-s001.docx]

**Table S1. Comparison of Study Variables betw5een ER and LR Groups across Two Independent Institutions (N = 225)**

| Variable | Institution 1 (n=181) | | | | | | Institution 2 (n=44) | | |
| --- | --- | --- | --- | --- | --- | --- | --- | --- | --- |
|  | LR (n=137) | ER (n=44) | P-value | | LR (n=36) | | | ER (n=8) | P-value |
| **Demographic Characteristics** | | | | | | | | | |
| Age, years | 62.00 [55.00, 69.00] | 63.50 [59.00, 66.25] | 0.37 | | 62.50 [56.75, 69.00] | | | 64.00 [60.50, 68.75] | 0.57 |
| Sex, male/female | 74/63 | 22/22 | 0.77 | | 18/18 | | | 7/1 | 0.11 |
| BMI, kg/m² | 23.44 [21.19, 25.21] | 22.49 [21.26, 25.49] | 0.97 | | 22.37 [21.27, 24.67] | | | 21.87 [20.39, 23.72] | 0.36 |
| **Clinical Characteristics** | | | | | | | | | |
| Smoking history, no/yes | 93/22 | 27/11 | 0.41 | | 26/10 | | | 5/3 | 0.68 |
| Drinking history, no/yes | 92/35 | 30/8 | 0.38 | | 26/10 | | | 5/3 | 0.68 |
| Diabetes, no/yes | 96/41 | 30/14 | 0.96 | | 24/12 | | | 4/4 | 0.43 |
| Hypertension, no/yes | 79/58 | 24/20 | 0.85 | | 20/16 | | | 2/6 | 0.24 |
| Hyperlipidemia, no/yes | 119/18 | 44/0 | 0.01 | | 31/5 | | | 7/1 | 1.00 |
| Pancreatitis, no/yes | 120/9 | 38/4 | 0.82 | | 32/4 | | | 6/2 | 0.30 |
| Pancreatic cancer family history, no/yes | 129/8 | 43/1 | 0.69 | | 33/3 | | | 8/0 | 1.00 |
| **Laboratory Parameters** | | | | | | | | | |
| LYMPH, % | 28.10 [23.20, 32.80] | 25.45 [20.65, 30.50] | 0.20 | | 26.60 [21.38, 30.75] | | | 30.75 [27.80, 36.35] | 0.01 |
| MONO, % | 6.20 [5.10, 7.00] | 6.10 [5.07, 7.30] | 0.93 | | 6.60 [5.88, 7.33] | | | 6.70 [5.05, 7.67] | 0.96 |
| NEUT, % | 60.90 [56.00, 66.40] | 63.00 [58.18, 70.40] | 0.14 | | 64.30 [58.98, 67.78] | | | 60.25 [52.10, 63.85] | 0.02 |
| ALT, U/L | 19.00 [13.00, 41.00] | 21.00 [12.75, 58.75] | 0.74 | | 26.50 [16.75, 151.75] | | | 33.50 [29.00, 52.00] | 0.83 |
| AST, U/L | 19.00 [16.00, 32.00] | 22.00 [15.00, 47.75] | 0.68 | | 23.00 [19.00, 79.75] | | | 27.00 [22.00, 45.00] | 0.55 |
| TP, g/L | 68.00 [64.00, 71.00] | 67.00 [63.00, 70.00] | 0.23 | | 66.50 [62.22, 71.65] | | | 68.00 [57.50, 72.05] | 0.38 |
| ALB, g/L | 42.00 [40.00, 44.00] | 41.00 [38.00, 43.00] | 0.06 | | 41.00 [39.00, 44.92] | | | 41.50 [38.58, 44.03] | 0.57 |
| TBIL, μmol/L | 12.60 [9.50, 20.80] | 13.60 [11.00, 53.95] | 0.13 | | 16.50 [11.38, 67.90] | | | 16.80 [11.00, 231.40] | 0.86 |
| GGT, U/L | 23.00 [16.00, 95.00] | 29.50 [16.00, 165.00] | 0.32 | | 39.00 [22.00, 226.75] | | | 59.00 [38.00, 138.00] | 0.34 |
| TBA, μmol/L | 3.60 [2.12, 6.88] | 2.75 [1.82, 12.70] | 0.89 | | 4.30 [2.35, 11.97] | | | 4.00 [2.75, 5.25] | 0.58 |
| GLU, mmol/L | 6.00 [5.30, 7.20] | 6.65 [5.57, 8.05] | 0.04 | | 6.15 [5.38, 7.72] | | | 7.20 [6.09, 8.22] | 0.22 |
| TG, mmol/L | 1.20 [0.89, 1.71] | 1.21 [1.06, 1.80] | 0.27 | | 1.29 [0.91, 1.49] | | | 2.52 [1.17, 3.49] | 0.08 |
| CRP, mg/L | 1.29 [0.69, 2.99] | 2.01 [0.92, 7.68] | 0.02 | | 1.97 [0.57, 4.31] | | | 3.76 [2.30, 38.18] | 0.34 |
| CA199, U/mL | 101.00 [34.80, 327.00] | 197.00 [88.50, 481.35] | 0.02 | | 122.00 [31.40, 220.50] | | | 156.65 [56.77, 452.75] | 0.46 |
| CEA, μg/L | 3.00 [1.60, 4.15] | 3.10 [2.08, 4.77] | 0.15 | | 2.40 [1.65, 3.55] | | | 2.61 [2.02, 4.05] | 0.48 |
| CA125, U/mL | 12.10 [8.15, 20.00] | 14.00 [8.80, 20.90] | 0.37 | | 9.50 [5.90, 13.70] | | | 14.80 [10.75, 28.55] | 0.29 |
| **Pathological Characteristics** | | | | | | | | | |
| Max Diameter, cm | 3.00 [2.10, 4.00] | 3.10 [2.50, 4.05] | 0.192 | | 3.00 [2.48, 3.50] | | | 2.75 [1.80, 3.25] | 0.48 |
| Tumor location, head/body/tail/whole | 74/28/34/1 | 21/8/13/2 | 0.31 | | 22/9/5/0 | | | 7/1/0/0 | 0.32 |
| Peripheral invasion, no/yes | 77/60 | 21/23 | 0.42 | | 18/18 | | | 3/5 | 0.70 |
| Cell differentiation, poor/moderate/well | 42/85/10 | 22/22/0 | 0.02 | | 11/25/0 | | | 2/6/0 | 1.00 |
| Lymph node metastasis, no/yes | 68/69 | 27/17 | 0.24 | | 20/16 | | | 5/3 | 1.00 |
| T stage, T1/T2/T3/T4 | 32/74/26/5 | 7/17/15/5 | 0.02 | | 8/22/4/2 | | | 3/3/2/0 | 0.45 |
| N stage, N0/N1/N2 | 69/57/11 | 17/17/10 | 0.03 | | 20/14/2 | | | 5/3/0 | 0.78 |
| **Treatment** | | | | | | | | | |
| Adjuvant Chemo (GS/Platinum/AG/Others/Unknown) | 53/9/11/30/34 | 14/2/7/14/7 | | 0.26 | | 10/3/6/9/8 | | 1/0/3/2/2 | 0.61 |
| **Survival Outcomes** | | | | | | | | | |
| Median RFS, months | 26.67 [11.93, 41.73] | 3.83 [2.21, 5.02] | ＜0.01 | | 32.95 [16.49, 41.22] | | | 2.42 [1.47, 3.93] | ＜0.01 |

**Note:** Data are presented as Mean ± Standard deviation or Median [25th Percentile, 75th Percentile] for continuous variables and counts for categorical variables. P-values for continuous variables were calculated using one-way analysis of variance or Kruskal-Wallis H test; for categorical variables, Pearson's chi-square test (χ²) or Fisher's exact test (Fisher's exact) was used as appropriate. Abbreviations: χ², Chi-square statistic; df, Degrees of freedom; BMI, Body Mass Index; LYMPH, lymphocyte; MONO, monocyte; NEUT, neutrophil; ALT, alanine aminotransferase; AST, aspartate aminotransferase; TP, total protein; ALB, albumin; TBIL, total bilirubin; GGT, gamma-glutamyl transferase; TBA, total bile acid; GLU, glucose; TG, triglycerides; CRP, C-reactive protein; CEA, carcinoembryonic antigen; GS, gemcitabine plus S-1; Platinum, platinum-based regimens; AG, nab-paclitaxel plus gemcitabine; RFS, recurrence-free survival.

**Table S2. Performance of the Rad-Path models on training set.**

| Model | AUC | Accuracy | Balanced Accuracy | F1-score | Sensitivity | Specificity | PPV | NPV |
| --- | --- | --- | --- | --- | --- | --- | --- | --- |
| AB | 0.964 [0.934-0.986] | 0.683 [0.606-0.761] | 0.784 [0.734-0.830] | 0.628 [0.514-0.721] | 1.000 [1.000-1.000] | 0.567 [0.467-0.660] | 0.458 [0.346-0.563] | 1.000 [1.000-1.000] |
| BN | 0.640 [0.527-0.739] | 0.697 [0.627-0.768] | 0.618 [0.531-0.705] | 0.442 [0.290-0.571] | 0.447 [0.286-0.611] | 0.788 [0.709-0.865] | 0.436 [0.270-0.600] | 0.796 [0.720-0.870] |
| DT | 0.861 [0.808-0.909] | 0.782 [0.718-0.845] | 0.801 [0.731-0.869] | 0.674 [0.558-0.778] | 0.842 [0.727-0.952] | 0.760 [0.673-0.840] | 0.561 [0.433-0.692] | 0.929 [0.872-0.978] |
| GB | 1.000 [1.000-1.000] | 0.887 [0.831-0.937] | 0.923 [0.887-0.957] | 0.826 [0.733-0.903] | 1.000 [1.000-1.000] | 0.846 [0.774-0.914] | 0.704 [0.579-0.823] | 1.000 [1.000-1.000] |
| GN | 0.716 [0.624-0.797] | 0.479 [0.401-0.556] | 0.611 [0.539-0.673] | 0.479 [0.373-0.569] | 0.895 [0.781-0.976] | 0.327 [0.243-0.417] | 0.327 [0.241-0.412] | 0.895 [0.783-0.976] |
| KNN | 0.981 [0.963-0.996] | 0.789 [0.718-0.852] | 0.856 [0.810-0.898] | 0.717 [0.610-0.803] | 1.000 [1.000-1.000] | 0.712 [0.620-0.796] | 0.559 [0.439-0.671] | 1.000 [1.000-1.000] |
| LDA | 0.636 [0.527-0.737] | 0.289 [0.211-0.366] | 0.514 [0.500-0.535] | 0.429 [0.331-0.521] | 1.000 [1.000-1.000] | 0.029 [0.000-0.069] | 0.273 [0.199-0.353] | 1.000 [0.000-1.000] |
| LRG | 0.634 [0.526-0.729] | 0.303 [0.225-0.380] | 0.524 [0.505-0.546] | 0.434 [0.337-0.524] | 1.000 [1.000-1.000] | 0.048 [0.010-0.092] | 0.277 [0.203-0.355] | 1.000 [1.000-1.000] |
| RF | 1.000 [1.000-1.000] | 1.000 [1.000-1.000] | 1.000 [1.000-1.000] | 1.000 [1.000-1.000] | 1.000 [1.000-1.000] | 1.000 [1.000-1.000] | 1.000 [1.000-1.000] | 1.000 [1.000-1.000] |
| SVM | 0.982 [0.963-0.995] | 0.873 [0.817-0.930] | 0.913 [0.876-0.948] | 0.809 [0.714-0.891] | 1.000 [1.000-1.000] | 0.827 [0.752-0.897] | 0.679 [0.556-0.803] | 1.000 [1.000-1.000] |
| XGB | 1.000 [1.000-1.000] | 1.000 [1.000-1.000] | 1.000 [1.000-1.000] | 1.000 [1.000-1.000] | 1.000 [1.000-1.000] | 1.000 [1.000-1.000] | 1.000 [1.000-1.000] | 1.000 [1.000-1.000] |

**Note:** Performance metrics are presented as the AUC with 95% confidence intervals in square brackets. Abbreviations: AB = Adaptive Boosting, BN = Bernoulli NB, DT = Decision Tree, GB = Gradient Boosting, GN = Gaussian NB, K Nearest Neighbors = KNN, Linear Discriminant Analysis = LDA, Logistic Regression = LRG, Random Forest = RF, SVM = Support Vector Machine, XGB = XG Boost, AUC = Area Under the Curve, NPV = Negative Predictive Value, PPV = Positive Predictive Value.

**Table S3. Performance of the Rad-Path models on internal test set.**

| Model | AUC | Accuracy | Balanced Accuracy | F1-score | Sensitivity | Specificity | PPV | NPV |
| --- | --- | --- | --- | --- | --- | --- | --- | --- |
| AB | 0.783 [0.486-0.981] | 0.718 [0.564-0.846] | 0.765 [0.564-0.903] | 0.476 [0.167-0.714] | 0.833 [0.500-1.000] | 0.697 [0.543-0.844] | 0.333 [0.100-0.583] | 0.958 [0.867-1.000] |
| BN | 0.621 [0.344-0.848] | 0.667 [0.513-0.821] | 0.598 [0.371-0.851] | 0.316 [0.000-0.581] | 0.500 [0.000-1.000] | 0.697 [0.531-0.848] | 0.231 [0.000-0.471] | 0.885 [0.742-1.000] |
| DT | 0.692 [0.481-0.871] | 0.615 [0.462-0.769] | 0.705 [0.521-0.847] | 0.400 [0.143-0.600] | 0.833 [0.500-1.000] | 0.576 [0.400-0.743] | 0.263 [0.083-0.455] | 0.950 [0.842-1.000] |
| GB | 0.758 [0.552-0.917] | 0.615 [0.462-0.769] | 0.773 [0.690-0.855] | 0.444 [0.190-0.645] | 1.000 [1.000-1.000] | 0.545 [0.379-0.710] | 0.286 [0.105-0.476] | 1.000 [1.000-1.000] |
| GN | 0.616 [0.221-0.954] | 0.359 [0.231-0.513] | 0.485 [0.254-0.688] | 0.242 [0.065-0.444] | 0.667 [0.250-1.000] | 0.303 [0.147-0.471] | 0.148 [0.037-0.300] | 0.833 [0.571-1.000] |
| KNN | 0.672 [0.397-0.929] | 0.615 [0.462-0.769] | 0.636 [0.397-0.847] | 0.348 [0.095-0.583] | 0.667 [0.200-1.000] | 0.606 [0.452-0.781] | 0.235 [0.059-0.462] | 0.909 [0.765-1.000] |
| LDA | 0.455 [0.162-0.757] | 0.154 [0.051-0.282] | 0.500 [0.500-0.500] | 0.267 [0.098-0.440] | 1.000 [1.000-1.000] | 0.000 [0.000-0.000] | 0.154 [0.051-0.282] | 0.000 [0.000-0.000] |
| LRG | 0.465 [0.172-0.754] | 0.154 [0.051-0.282] | 0.500 [0.500-0.500] | 0.267 [0.098-0.440] | 1.000 [1.000-1.000] | 0.000 [0.000-0.000] | 0.154 [0.051-0.282] | 0.000 [0.000-0.000] |
| RF | 0.851 [0.694-0.965] | 0.795 [0.667-0.897] | 0.674 [0.429-0.929] | 0.429 [0.000-0.714] | 0.500 [0.000-1.000] | 0.848 [0.719-0.944] | 0.375 [0.000-0.750] | 0.903 [0.786-1.000] |
| SVM | 0.571 [0.333-0.824] | 0.538 [0.385-0.667] | 0.523 [0.286-0.781] | 0.250 [0.000-0.483] | 0.500 [0.000-1.000] | 0.545 [0.375-0.714] | 0.167 [0.000-0.353] | 0.857 [0.682-1.000] |
| XGB | 0.773 [0.611-0.907] | 0.744 [0.590-0.872] | 0.576 [0.378-0.779] | 0.286 [0.000-0.556] | 0.333 [0.000-0.750] | 0.818 [0.676-0.941] | 0.250 [0.000-0.600] | 0.871 [0.733-0.969] |

**Note:** Performance metrics are presented as the AUC with 95% confidence intervals in square brackets. Abbreviations: AB = Adaptive Boosting, BN = Bernoulli NB, DT = Decision Tree, GB = Gradient Boosting, GN = Gaussian NB, K Nearest Neighbors = KNN, Linear Discriminant Analysis = LDA, Logistic Regression = LRG, Random Forest = RF, SVM = Support Vector Machine, XGB = XG Boost, AUC = Area Under the Curve, NPV = Negative Predictive Value, PPV = Positive Predictive Value.

**Table S4. Performance of the Rad-Path models on external test set.**

| Model | AUC | Accuracy | Balanced Accuracy | F1-score | Sensitivity | Specificity | PPV | NPV |
| --- | --- | --- | --- | --- | --- | --- | --- | --- |
| AB | 0.622 [0.431-0.799] | 0.432 [0.295-0.568] | 0.604 [0.447-0.732] | 0.359 [0.167-0.542] | 0.875 [0.600-1.000] | 0.333 [0.194-0.486] | 0.226 [0.094-0.379] | 0.923 [0.750-1.000] |
| BN | 0.743 [0.522-0.927] | 0.682 [0.545-0.818] | 0.660 [0.456-0.851] | 0.417 [0.125-0.645] | 0.625 [0.222-1.000] | 0.694 [0.538-0.842] | 0.312 [0.091-0.571] | 0.893 [0.778-1.000] |
| DT | 0.616 [0.441-0.757] | 0.523 [0.364-0.659] | 0.562 [0.344-0.744] | 0.323 [0.083-0.513] | 0.625 [0.231-1.000] | 0.500 [0.324-0.657] | 0.217 [0.050-0.381] | 0.857 [0.688-1.000] |
| GB | 0.819 [0.691-0.942] | 0.591 [0.455-0.727] | 0.750 [0.662-0.833] | 0.471 [0.235-0.653] | 1.000 [1.000-1.000] | 0.500 [0.324-0.667] | 0.308 [0.133-0.485] | 1.000 [1.000-1.000] |
| GN | 0.618 [0.361-0.850] | 0.273 [0.136-0.409] | 0.507 [0.360-0.618] | 0.304 [0.118-0.473] | 0.875 [0.600-1.000] | 0.139 [0.029-0.256] | 0.184 [0.062-0.314] | 0.833 [0.500-1.000] |
| KNN | 0.623 [0.426-0.814] | 0.409 [0.273-0.545] | 0.590 [0.435-0.711] | 0.350 [0.162-0.524] | 0.875 [0.600-1.000] | 0.306 [0.158-0.457] | 0.219 [0.094-0.364] | 0.917 [0.714-1.000] |
| LDA | 0.538 [0.309-0.780] | 0.182 [0.068-0.295] | 0.500 [0.500-0.500] | 0.308 [0.128-0.456] | 1.000 [1.000-1.000] | 0.000 [0.000-0.000] | 0.182 [0.068-0.295] | 0.000 [0.000-0.000] |
| LRG | 0.552 [0.328-0.766] | 0.182 [0.091-0.295] | 0.500 [0.500-0.500] | 0.308 [0.167-0.456] | 1.000 [1.000-1.000] | 0.000 [0.000-0.000] | 0.182 [0.091-0.295] | 0.000 [0.000-0.000] |
| RF | 0.814 [0.677-0.934] | 0.750 [0.614-0.886] | 0.701 [0.488-0.895] | 0.476 [0.154-0.727] | 0.625 [0.250-1.000] | 0.778 [0.641-0.909] | 0.385 [0.111-0.667] | 0.903 [0.786-1.000] |
| SVM | 0.543 [0.344-0.762] | 0.545 [0.409-0.682] | 0.576 [0.381-0.771] | 0.333 [0.091-0.541] | 0.625 [0.250-1.000] | 0.528 [0.371-0.686] | 0.227 [0.056-0.429] | 0.864 [0.708-1.000] |
| XGB | 0.767 [0.600-0.923] | 0.636 [0.500-0.773] | 0.681 [0.498-0.841] | 0.429 [0.182-0.629] | 0.750 [0.400-1.000] | 0.611 [0.450-0.763] | 0.300 [0.105-0.500] | 0.917 [0.781-1.000] |

**Note:** Performance metrics are presented as the AUC with 95% confidence intervals in square brackets. Abbreviations: AB = Adaptive Boosting, BN = Bernoulli NB, DT = Decision Tree, GB = Gradient Boosting, GN = Gaussian NB, K Nearest Neighbors = KNN, Linear Discriminant Analysis = LDA, Logistic Regression = LRG, Random Forest = RF, SVM = Support Vector Machine, XGB = XG Boost, AUC = Area Under the Curve, NPV = Negative Predictive Value, PPV = Positive Predictive Value.

**Table S5. Performance of the tumor-region radiomics models on training set.**

| Model | AUC | Accuracy | Balanced Accuracy | F1-score | Sensitivity | Specificity | PPV | NPV |
| --- | --- | --- | --- | --- | --- | --- | --- | --- |
| AB | 0.821 [0.735-0.889] | 0.782 [0.711-0.845] | 0.717 [0.623-0.801] | 0.587 [0.429-0.708] | 0.579 [0.406-0.732] | 0.856 [0.784-0.922] | 0.595 [0.417-0.750] | 0.848 [0.780-0.914] |
| BN | 0.691 [0.592-0.790] | 0.669 [0.592-0.739] | 0.649 [0.565-0.733] | 0.495 [0.369-0.614] | 0.605 [0.452-0.757] | 0.692 [0.600-0.775] | 0.418 [0.294-0.556] | 0.828 [0.742-0.899] |
| DT | 0.697 [0.602-0.786] | 0.775 [0.711-0.845] | 0.688 [0.602-0.772] | 0.543 [0.387-0.675] | 0.500 [0.333-0.667] | 0.875 [0.812-0.934] | 0.594 [0.419-0.758] | 0.827 [0.757-0.900] |
| GB | 0.746 [0.652-0.837] | 0.599 [0.514-0.676] | 0.676 [0.603-0.747] | 0.529 [0.417-0.632] | 0.842 [0.724-0.950] | 0.510 [0.415-0.604] | 0.386 [0.284-0.494] | 0.898 [0.821-0.966] |
| GN | 1.000 [1.000-1.000] | 0.986 [0.965-1.000] | 0.990 [0.974-1.000] | 0.974 [0.930-1.000] | 1.000 [1.000-1.000] | 0.981 [0.949-1.000] | 0.950 [0.868-1.000] | 1.000 [1.000-1.000] |
| KNN | 0.771 [0.687-0.847] | 0.521 [0.437-0.599] | 0.673 [0.630-0.719] | 0.528 [0.419-0.617] | 1.000 [1.000-1.000] | 0.346 [0.260-0.438] | 0.358 [0.265-0.447] | 1.000 [1.000-1.000] |
| LDA | 0.739 [0.635-0.835] | 0.676 [0.599-0.754] | 0.695 [0.610-0.776] | 0.549 [0.420-0.660] | 0.737 [0.591-0.872] | 0.654 [0.561-0.747] | 0.438 [0.315-0.565] | 0.872 [0.788-0.944] |
| LRG | 0.678 [0.566-0.783] | 0.761 [0.683-0.824] | 0.628 [0.544-0.706] | 0.433 [0.261-0.576] | 0.342 [0.188-0.500] | 0.913 [0.851-0.963] | 0.591 [0.353-0.808] | 0.792 [0.713-0.858] |
| RF | 0.929 [0.878-0.968] | 0.873 [0.817-0.923] | 0.855 [0.783-0.920] | 0.775 [0.667-0.873] | 0.816 [0.682-0.935] | 0.894 [0.835-0.948] | 0.738 [0.605-0.867] | 0.930 [0.877-0.979] |
| SVM | 0.936 [0.889-0.978] | 0.866 [0.803-0.923] | 0.808 [0.724-0.886] | 0.732 [0.595-0.844] | 0.684 [0.529-0.837] | 0.933 [0.879-0.979] | 0.788 [0.639-0.920] | 0.890 [0.826-0.947] |
| XGB | 0.743 [0.638-0.837] | 0.690 [0.613-0.768] | 0.688 [0.603-0.773] | 0.542 [0.411-0.661] | 0.684 [0.532-0.829] | 0.692 [0.607-0.780] | 0.448 [0.318-0.576] | 0.857 [0.775-0.928] |

**Note:** Performance metrics are presented as the AUC with 95% confidence intervals in square brackets. Abbreviations: AB = Adaptive Boosting, BN = Bernoulli NB, DT = Decision Tree, GB = Gradient Boosting, GN = Gaussian NB, K Nearest Neighbors = KNN, Linear Discriminant Analysis = LDA, Logistic Regression = LRG, Random Forest = RF, SVM = Support Vector Machine, XGB = XG Boost, AUC = Area Under the Curve, NPV = Negative Predictive Value, PPV = Positive Predictive Value.

**Table S6. Performance of the tumor-region radiomicsmodels on internal test set.**

| Model | AUC | Accuracy | Balanced Accuracy | F1-score | Sensitivity | Specificity | PPV | NPV |
| --- | --- | --- | --- | --- | --- | --- | --- | --- |
| AB | 0.780 [0.549-0.967] | 0.795 [0.667-0.923] | 0.674 [0.444-0.924] | 0.429 [0.000-0.727] | 0.500 [0.000-1.000] | 0.848 [0.719-0.969] | 0.375 [0.000-0.750] | 0.903 [0.781-1.000] |
| BN | 0.753 [0.509-0.948] | 0.641 [0.487-0.795] | 0.720 [0.494-0.875] | 0.417 [0.111-0.667] | 0.833 [0.400-1.000] | 0.606 [0.438-0.781] | 0.278 [0.062-0.500] | 0.952 [0.842-1.000] |
| DT | 0.674 [0.434-0.926] | 0.769 [0.641-0.897] | 0.659 [0.409-0.926] | 0.400 [0.000-0.667] | 0.500 [0.000-1.000] | 0.818 [0.667-0.938] | 0.333 [0.000-0.667] | 0.900 [0.783-1.000] |
| GB | 0.712 [0.352-0.973] | 0.513 [0.359-0.641] | 0.576 [0.343-0.786] | 0.296 [0.080-0.514] | 0.667 [0.250-1.000] | 0.485 [0.312-0.645] | 0.190 [0.045-0.381] | 0.889 [0.722-1.000] |
| GN | 0.697 [0.417-0.939] | 0.744 [0.590-0.872] | 0.576 [0.375-0.811] | 0.286 [0.000-0.571] | 0.333 [0.000-0.750] | 0.818 [0.667-0.935] | 0.250 [0.000-0.600] | 0.871 [0.733-0.970] |
| KNN | 0.548 [0.253-0.806] | 0.410 [0.256-0.564] | 0.515 [0.258-0.714] | 0.258 [0.065-0.444] | 0.667 [0.200-1.000] | 0.364 [0.206-0.529] | 0.160 [0.036-0.308] | 0.857 [0.643-1.000] |
| LDA | 0.677 [0.421-0.905] | 0.641 [0.487-0.769] | 0.652 [0.411-0.853] | 0.364 [0.100-0.600] | 0.667 [0.200-1.000] | 0.636 [0.457-0.800] | 0.250 [0.059-0.467] | 0.913 [0.773-1.000] |
| LRG | 0.672 [0.417-0.900] | 0.795 [0.667-0.897] | 0.606 [0.417-0.841] | 0.333 [0.000-0.667] | 0.333 [0.000-0.800] | 0.879 [0.758-0.971] | 0.333 [0.000-0.800] | 0.879 [0.750-0.971] |
| RF | 0.788 [0.563-0.953] | 0.615 [0.462-0.769] | 0.705 [0.507-0.853] | 0.400 [0.111-0.615] | 0.833 [0.500-1.000] | 0.576 [0.406-0.735] | 0.263 [0.062-0.471] | 0.950 [0.833-1.000] |
| SVM | 0.667 [0.361-0.907] | 0.744 [0.615-0.872] | 0.576 [0.379-0.804] | 0.286 [0.000-0.556] | 0.333 [0.000-0.750] | 0.818 [0.676-0.938] | 0.250 [0.000-0.600] | 0.871 [0.758-0.970] |
| XGB | 0.692 [0.459-0.900] | 0.667 [0.513-0.795] | 0.598 [0.371-0.842] | 0.316 [0.000-0.571] | 0.500 [0.000-1.000] | 0.697 [0.543-0.844] | 0.231 [0.000-0.500] | 0.885 [0.750-1.000] |

**Note:** Performance metrics are presented as the AUC with 95% confidence intervals in square brackets. Abbreviations: AB = Adaptive Boosting, BN = Bernoulli NB, DT = Decision Tree, GB = Gradient Boosting, GN = Gaussian NB, K Nearest Neighbors = KNN, Linear Discriminant Analysis = LDA, Logistic Regression = LRG, Random Forest = RF, SVM = Support Vector Machine, XGB = XG Boost, AUC = Area Under the Curve, NPV = Negative Predictive Value, PPV = Positive Predictive Value.

**Table S7. Performance of the tumor-region radiomics models on external test set.**

| Model | AUC | Accuracy | Balanced Accuracy | F1-score | Sensitivity | Specificity | PPV | NPV |
| --- | --- | --- | --- | --- | --- | --- | --- | --- |
| AB | 0.658 [0.407-0.871] | 0.659 [0.523-0.795] | 0.549 [0.354-0.758] | 0.286 [0.000-0.552] | 0.375 [0.000-0.750] | 0.722 [0.571-0.865] | 0.231 [0.000-0.500] | 0.839 [0.700-0.962] |
| BN | 0.521 [0.316-0.756] | 0.682 [0.545-0.818] | 0.562 [0.371-0.775] | 0.300 [0.000-0.556] | 0.375 [0.000-0.750] | 0.750 [0.606-0.879] | 0.250 [0.000-0.538] | 0.844 [0.710-0.968] |
| DT | 0.660 [0.464-0.843] | 0.705 [0.568-0.841] | 0.625 [0.421-0.825] | 0.381 [0.100-0.632] | 0.500 [0.143-0.875] | 0.750 [0.606-0.882] | 0.308 [0.071-0.583] | 0.871 [0.742-0.971] |
| GB | 0.500 [0.231-0.768] | 0.591 [0.432-0.750] | 0.604 [0.417-0.803] | 0.357 [0.118-0.571] | 0.625 [0.250-1.000] | 0.583 [0.421-0.765] | 0.250 [0.074-0.450] | 0.875 [0.724-1.000] |
| GN | 0.649 [0.388-0.877] | 0.705 [0.568-0.841] | 0.625 [0.413-0.811] | 0.381 [0.100-0.625] | 0.500 [0.125-0.833] | 0.750 [0.610-0.886] | 0.308 [0.067-0.588] | 0.871 [0.742-0.969] |
| KNN | 0.365 [0.193-0.550] | 0.341 [0.205-0.477] | 0.451 [0.259-0.634] | 0.256 [0.065-0.432] | 0.625 [0.250-1.000] | 0.278 [0.143-0.429] | 0.161 [0.038-0.297] | 0.769 [0.500-1.000] |
| LDA | 0.462 [0.219-0.718] | 0.545 [0.386-0.682] | 0.479 [0.280-0.680] | 0.231 [0.000-0.452] | 0.375 [0.000-0.750] | 0.583 [0.429-0.750] | 0.167 [0.000-0.364] | 0.808 [0.640-0.957] |
| LRG | 0.642 [0.410-0.847] | 0.727 [0.591-0.841] | 0.493 [0.382-0.651] | 0.143 [0.000-0.400] | 0.125 [0.000-0.400] | 0.861 [0.742-0.971] | 0.167 [0.000-0.500] | 0.816 [0.683-0.927] |
| RF | 0.613 [0.367-0.855] | 0.659 [0.523-0.795] | 0.646 [0.450-0.841] | 0.400 [0.111-0.621] | 0.625 [0.250-1.000] | 0.667 [0.512-0.818] | 0.294 [0.071-0.529] | 0.889 [0.750-1.000] |
| SVM | 0.392 [0.138-0.662] | 0.750 [0.614-0.864] | 0.507 [0.403-0.646] | 0.154 [0.000-0.421] | 0.125 [0.000-0.400] | 0.889 [0.784-0.974] | 0.200 [0.000-0.667] | 0.821 [0.690-0.927] |
| XGB | 0.469 [0.210-0.713] | 0.523 [0.386-0.659] | 0.417 [0.256-0.606] | 0.160 [0.000-0.357] | 0.250 [0.000-0.600] | 0.583 [0.424-0.737] | 0.118 [0.000-0.286] | 0.778 [0.621-0.923] |

**Note:** Performance metrics are presented as the AUC with 95% confidence intervals in square brackets. Abbreviations: AB = Adaptive Boosting, BN = Bernoulli NB, DT = Decision Tree, GB = Gradient Boosting, GN = Gaussian NB, K Nearest Neighbors = KNN, Linear Discriminant Analysis = LDA, Logistic Regression = LRG, Random Forest = RF, SVM = Support Vector Machine, XGB = XG Boost, AUC = Area Under the Curve, NPV = Negative Predictive Value, PPV = Positive Predictive Value.

**Table S8. Performance of the peritumoral-region radiomics models on training set.**

| Model | AUC | Accuracy | Balanced Accuracy | F1-score | Sensitivity | Specificity | PPV | NPV |
| --- | --- | --- | --- | --- | --- | --- | --- | --- |
| AB | 0.786 [0.712-0.857] | 0.796 [0.732-0.866] | 0.710 [0.627-0.795] | 0.580 [0.418-0.704] | 0.526 [0.359-0.681] | 0.894 [0.832-0.951] | 0.645 [0.462-0.808] | 0.838 [0.769-0.905] |
| BN | 0.685 [0.582-0.785] | 0.697 [0.627-0.761] | 0.643 [0.555-0.726] | 0.482 [0.351-0.607] | 0.526 [0.368-0.683] | 0.760 [0.673-0.836] | 0.444 [0.311-0.595] | 0.814 [0.736-0.885] |
| DT | 0.794 [0.720-0.863] | 0.824 [0.768-0.887] | 0.738 [0.653-0.824] | 0.627 [0.480-0.754] | 0.553 [0.395-0.714] | 0.923 [0.870-0.971] | 0.724 [0.560-0.880] | 0.850 [0.786-0.915] |
| GB | 0.749 [0.656-0.833] | 0.725 [0.648-0.803] | 0.629 [0.540-0.721] | 0.451 [0.296-0.588] | 0.421 [0.262-0.576] | 0.837 [0.760-0.909] | 0.485 [0.314-0.667] | 0.798 [0.725-0.875] |
| GN | 0.986 [0.966-0.999] | 0.958 [0.923-0.986] | 0.954 [0.910-0.990] | 0.923 [0.857-0.977] | 0.947 [0.865-1.000] | 0.962 [0.922-0.991] | 0.900 [0.800-0.977] | 0.980 [0.951-1.000] |
| KNN | 1.000 [1.000-1.000] | 1.000 [1.000-1.000] | 1.000 [1.000-1.000] | 1.000 [1.000-1.000] | 1.000 [1.000-1.000] | 1.000 [1.000-1.000] | 1.000 [1.000-1.000] | 1.000 [1.000-1.000] |
| LDA | 0.586 [0.470-0.691] | 0.732 [0.655-0.803] | 0.500 [0.500-0.500] | 0.000 [0.000-0.000] | 0.000 [0.000-0.000] | 1.000 [1.000-1.000] | 0.000 [0.000-0.000] | 0.732 [0.655-0.803] |
| LRG | 0.556 [0.440-0.669] | 0.732 [0.655-0.803] | 0.500 [0.500-0.500] | 0.000 [0.000-0.000] | 0.000 [0.000-0.000] | 1.000 [1.000-1.000] | 0.000 [0.000-0.000] | 0.732 [0.655-0.803] |
| RF | 1.000 [1.000-1.000] | 1.000 [1.000-1.000] | 1.000 [1.000-1.000] | 1.000 [1.000-1.000] | 1.000 [1.000-1.000] | 1.000 [1.000-1.000] | 1.000 [1.000-1.000] | 1.000 [1.000-1.000] |
| SVM | 0.897 [0.837-0.948] | 0.824 [0.761-0.887] | 0.771 [0.693-0.853] | 0.667 [0.543-0.783] | 0.658 [0.514-0.800] | 0.885 [0.824-0.945] | 0.676 [0.525-0.837] | 0.876 [0.804-0.936] |
| XGB | 1.000 [1.000-1.000] | 1.000 [1.000-1.000] | 1.000 [1.000-1.000] | 1.000 [1.000-1.000] | 1.000 [1.000-1.000] | 1.000 [1.000-1.000] | 1.000 [1.000-1.000] | 1.000 [1.000-1.000] |

**Note:** Performance metrics are presented as the AUC with 95% confidence intervals in square brackets. Abbreviations: AB = Adaptive Boosting, BN = Bernoulli NB, DT = Decision Tree, GB = Gradient Boosting, GN = Gaussian NB, K Nearest Neighbors = KNN, Linear Discriminant Analysis = LDA, Logistic Regression = LRG, Random Forest = RF, SVM = Support Vector Machine, XGB = XG Boost, AUC = Area Under the Curve, NPV = Negative Predictive Value, PPV = Positive Predictive Value.

**Table S9. Performance of the peritumoral-region radiomics models on internal test set.**

| Model | AUC | Accuracy | Balanced Accuracy | F1-score | Sensitivity | Specificity | PPV | NPV |
| --- | --- | --- | --- | --- | --- | --- | --- | --- |
| AB | 0.578 [0.346-0.815] | 0.692 [0.538-0.821] | 0.545 [0.348-0.774] | 0.250 [0.000-0.526] | 0.333 [0.000-0.750] | 0.758 [0.607-0.900] | 0.200 [0.000-0.500] | 0.862 [0.719-0.967] |
| BN | 0.510 [0.237-0.788] | 0.667 [0.513-0.821] | 0.530 [0.338-0.775] | 0.235 [0.000-0.522] | 0.333 [0.000-0.750] | 0.727 [0.576-0.879] | 0.182 [0.000-0.500] | 0.857 [0.708-0.967] |
| DT | 0.707 [0.514-0.886] | 0.795 [0.641-0.897] | 0.606 [0.412-0.832] | 0.333 [0.000-0.615] | 0.333 [0.000-0.778] | 0.879 [0.758-0.971] | 0.333 [0.000-0.750] | 0.879 [0.750-0.971] |
| GB | 0.424 [0.184-0.707] | 0.795 [0.667-0.923] | 0.538 [0.414-0.736] | 0.200 [0.000-0.571] | 0.167 [0.000-0.500] | 0.909 [0.800-1.000] | 0.250 [0.000-1.000] | 0.857 [0.730-0.971] |
| GN | 0.672 [0.429-0.886] | 0.692 [0.538-0.821] | 0.614 [0.375-0.853] | 0.333 [0.000-0.600] | 0.500 [0.000-1.000] | 0.727 [0.571-0.882] | 0.250 [0.000-0.545] | 0.889 [0.759-1.000] |
| KNN | 0.598 [0.312-0.888] | 0.641 [0.487-0.795] | 0.515 [0.317-0.750] | 0.222 [0.000-0.476] | 0.333 [0.000-0.750] | 0.697 [0.531-0.857] | 0.167 [0.000-0.400] | 0.852 [0.692-0.966] |
| LDA | 0.525 [0.136-0.941] | 0.846 [0.718-0.949] | 0.500 [0.500-0.500] | 0.000 [0.000-0.000] | 0.000 [0.000-0.000] | 1.000 [1.000-1.000] | 0.000 [0.000-0.000] | 0.846 [0.718-0.949] |
| LRG | 0.515 [0.081-0.926] | 0.846 [0.744-0.949] | 0.500 [0.500-0.500] | 0.000 [0.000-0.000] | 0.000 [0.000-0.000] | 1.000 [1.000-1.000] | 0.000 [0.000-0.000] | 0.846 [0.744-0.949] |
| RF | 0.568 [0.279-0.838] | 0.718 [0.564-0.846] | 0.629 [0.389-0.875] | 0.353 [0.000-0.615] | 0.500 [0.000-1.000] | 0.758 [0.600-0.903] | 0.273 [0.000-0.571] | 0.893 [0.767-1.000] |
| SVM | 0.424 [0.241-0.612] | 0.641 [0.487-0.795] | 0.379 [0.303-0.453] | 0.000 [0.000-0.000] | 0.000 [0.000-0.000] | 0.758 [0.606-0.906] | 0.000 [0.000-0.000] | 0.806 [0.656-0.933] |
| XGB | 0.495 [0.111-0.893] | 0.718 [0.564-0.846] | 0.629 [0.400-0.882] | 0.353 [0.000-0.625] | 0.500 [0.000-1.000] | 0.758 [0.606-0.886] | 0.273 [0.000-0.545] | 0.893 [0.769-1.000] |

**Note:** Performance metrics are presented as the AUC with 95% confidence intervals in square brackets. Abbreviations: AB = Adaptive Boosting, BN = Bernoulli NB, DT = Decision Tree, GB = Gradient Boosting, GN = Gaussian NB, K Nearest Neighbors = KNN, Linear Discriminant Analysis = LDA, Logistic Regression = LRG, Random Forest = RF, SVM = Support Vector Machine, XGB = XG Boost, AUC = Area Under the Curve, NPV = Negative Predictive Value, PPV = Positive Predictive Value.

**Table S10. Performance of the peritumoral-region radiomics models on external test set.**

| Model | AUC | Accuracy | Balanced Accuracy | F1-score | Sensitivity | Specificity | PPV | NPV |
| --- | --- | --- | --- | --- | --- | --- | --- | --- |
| AB | 0.556 [0.368-0.759] | 0.568 [0.432-0.705] | 0.444 [0.280-0.646] | 0.174 [0.000-0.387] | 0.250 [0.000-0.625] | 0.639 [0.485-0.784] | 0.133 [0.000-0.333] | 0.793 [0.645-0.933] |
| BN | 0.562 [0.307-0.822] | 0.591 [0.455-0.727] | 0.507 [0.312-0.718] | 0.250 [0.000-0.471] | 0.375 [0.000-0.750] | 0.639 [0.484-0.784] | 0.188 [0.000-0.400] | 0.821 [0.667-0.957] |
| DT | 0.538 [0.344-0.753] | 0.682 [0.523-0.818] | 0.514 [0.357-0.721] | 0.222 [0.000-0.500] | 0.250 [0.000-0.600] | 0.778 [0.629-0.914] | 0.200 [0.000-0.500] | 0.824 [0.690-0.941] |
| GB | 0.316 [0.069-0.615] | 0.773 [0.636-0.886] | 0.569 [0.425-0.759] | 0.286 [0.000-0.609] | 0.250 [0.000-0.600] | 0.889 [0.778-0.974] | 0.333 [0.000-0.750] | 0.842 [0.718-0.947] |
| GN | 0.486 [0.271-0.722] | 0.477 [0.341-0.636] | 0.389 [0.231-0.575] | 0.148 [0.000-0.333] | 0.250 [0.000-0.600] | 0.528 [0.375-0.694] | 0.105 [0.000-0.267] | 0.760 [0.600-0.923] |
| KNN | 0.688 [0.497-0.860] | 0.659 [0.523-0.795] | 0.597 [0.388-0.798] | 0.348 [0.091-0.593] | 0.500 [0.125-0.857] | 0.694 [0.545-0.844] | 0.267 [0.062-0.526] | 0.862 [0.720-0.967] |
| LDA | 0.427 [0.236-0.601] | 0.818 [0.705-0.932] | 0.500 [0.500-0.500] | 0.000 [0.000-0.000] | 0.000 [0.000-0.000] | 1.000 [1.000-1.000] | 0.000 [0.000-0.000] | 0.818 [0.705-0.932] |
| LRG | 0.490 [0.250-0.725] | 0.818 [0.705-0.909] | 0.500 [0.500-0.500] | 0.000 [0.000-0.000] | 0.000 [0.000-0.000] | 1.000 [1.000-1.000] | 0.000 [0.000-0.000] | 0.818 [0.705-0.909] |
| RF | 0.549 [0.372-0.730] | 0.568 [0.432-0.727] | 0.493 [0.300-0.708] | 0.240 [0.000-0.462] | 0.375 [0.000-0.750] | 0.611 [0.441-0.763] | 0.176 [0.000-0.375] | 0.815 [0.654-0.957] |
| SVM | 0.399 [0.194-0.648] | 0.659 [0.500-0.795] | 0.451 [0.338-0.620] | 0.118 [0.000-0.364] | 0.125 [0.000-0.444] | 0.778 [0.636-0.897] | 0.111 [0.000-0.375] | 0.800 [0.657-0.921] |
| XGB | 0.573 [0.368-0.746] | 0.591 [0.432-0.727] | 0.556 [0.346-0.746] | 0.308 [0.080-0.519] | 0.500 [0.143-0.857] | 0.611 [0.455-0.771] | 0.222 [0.050-0.429] | 0.846 [0.696-0.963] |

**Note:** Performance metrics are presented as the AUC with 95% confidence intervals in square brackets. Abbreviations: AB = Adaptive Boosting, BN = Bernoulli NB, DT = Decision Tree, GB = Gradient Boosting, GN = Gaussian NB, K Nearest Neighbors = KNN, Linear Discriminant Analysis = LDA, Logistic Regression = LRG, Random Forest = RF, SVM = Support Vector Machine, XGB = XG Boost, AUC = Area Under the Curve, NPV = Negative Predictive Value, PPV = Positive Predictive Value.

**Table S11. Performance of the pathological models on training set.**

| Model | AUC | ACC | Balanced Accuracy | F1-score | Sensitivity | Specificity | PPV | NPV |
| --- | --- | --- | --- | --- | --- | --- | --- | --- |
| AB | 0.602 [0.513-0.688] | 0.662 [0.585-0.739] | 0.602 [0.513-0.688] | 0.429 [0.289-0.551] | 0.474 [0.316-0.628] | 0.731 [0.647-0.821] | 0.391 [0.255-0.537] | 0.792 [0.713-0.867] |
| BN | 0.644 [0.552-0.740] | 0.380 [0.303-0.472] | 0.577 [0.545-0.613] | 0.463 [0.372-0.560] | 1.000 [1.000-1.000] | 0.154 [0.090-0.225] | 0.302 [0.228-0.388] | 1.000 [1.000-1.000] |
| DT | 0.703 [0.612-0.786] | 0.521 [0.437-0.606] | 0.640 [0.569-0.704] | 0.500 [0.400-0.604] | 0.895 [0.784-0.975] | 0.385 [0.289-0.480] | 0.347 [0.258-0.447] | 0.909 [0.816-0.979] |
| GB | 0.706 [0.610-0.793] | 0.585 [0.493-0.662] | 0.641 [0.549-0.718] | 0.496 [0.376-0.598] | 0.763 [0.629-0.889] | 0.519 [0.413-0.608] | 0.367 [0.260-0.476] | 0.857 [0.764-0.934] |
| GN | 1.000 [1.000-1.000] | 0.817 [0.746-0.880] | 0.875 [0.832-0.917] | 0.745 [0.639-0.833] | 1.000 [1.000-1.000] | 0.750 [0.664-0.835] | 0.594 [0.470-0.714] | 1.000 [1.000-1.000] |
| KNN | 0.835 [0.760-0.895] | 0.563 [0.486-0.648] | 0.685 [0.628-0.743] | 0.537 [0.421-0.638] | 0.947 [0.865-1.000] | 0.423 [0.336-0.522] | 0.375 [0.272-0.479] | 0.957 [0.886-1.000] |
| LDA | 0.695 [0.595-0.786] | 0.739 [0.662-0.810] | 0.572 [0.503-0.644] | 0.302 [0.133-0.464] | 0.211 [0.091-0.357] | 0.933 [0.882-0.979] | 0.533 [0.267-0.778] | 0.764 [0.683-0.837] |
| LRG | 0.637 [0.535-0.737] | 0.718 [0.648-0.789] | 0.566 [0.497-0.639] | 0.310 [0.154-0.456] | 0.237 [0.111-0.367] | 0.894 [0.832-0.950] | 0.450 [0.227-0.682] | 0.762 [0.688-0.838] |
| RF | 0.970 [0.942-0.991] | 0.585 [0.500-0.669] | 0.716 [0.673-0.765] | 0.563 [0.455-0.657] | 1.000 [1.000-1.000] | 0.433 [0.346-0.531] | 0.392 [0.295-0.490] | 1.000 [1.000-1.000] |
| SVM | 0.984 [0.960-0.999] | 0.951 [0.915-0.986] | 0.941 [0.891-0.983] | 0.909 [0.831-0.972] | 0.921 [0.825-1.000] | 0.962 [0.922-0.991] | 0.897 [0.788-0.976] | 0.971 [0.935-1.000] |
| XGB | 0.697 [0.599-0.790] | 0.746 [0.676-0.817] | 0.593 [0.515-0.665] | 0.357 [0.178-0.507] | 0.263 [0.118-0.405] | 0.923 [0.870-0.971] | 0.556 [0.308-0.778] | 0.774 [0.705-0.843] |

**Note:** Performance metrics are presented as the AUC with 95% confidence intervals in square brackets. Abbreviations: AB = Adaptive Boosting, BN = Bernoulli NB, DT = Decision Tree, GB = Gradient Boosting, GN = Gaussian NB, K Nearest Neighbors = KNN, Linear Discriminant Analysis = LDA, Logistic Regression = LRG, Random Forest = RF, SVM = Support Vector Machine, XGB = XG Boost, AUC = Area Under the Curve, NPV = Negative Predictive Value, PPV = Positive Predictive Value.

**Table S12. Performance of the pathological models on internal test set.**

| Model | AUC | Accuracy | Balanced Accuracy | F1-score | Sensitivity | Specificity | PPV | NPV |
| --- | --- | --- | --- | --- | --- | --- | --- | --- |
| AB | 0.386 [0.234-0.589] | 0.538 [0.385-0.692] | 0.386 [0.234-0.589] | 0.100 [0.000-0.296] | 0.167 [0.000-0.500] | 0.606 [0.438-0.771] | 0.071 [0.000-0.250] | 0.800 [0.640-0.950] |
| BN | 0.662 [0.321-0.899] | 0.256 [0.128-0.385] | 0.492 [0.280-0.618] | 0.256 [0.100-0.421] | 0.833 [0.429-1.000] | 0.152 [0.031-0.286] | 0.152 [0.053-0.278] | 0.833 [0.333-1.000] |
| DT | 0.652 [0.377-0.871] | 0.308 [0.154-0.462] | 0.523 [0.311-0.652] | 0.270 [0.065-0.450] | 0.833 [0.500-1.000] | 0.212 [0.083-0.353] | 0.161 [0.034-0.300] | 0.875 [0.600-1.000] |
| GB | 0.611 [0.311-0.857] | 0.513 [0.333-0.667] | 0.508 [0.250-0.757] | 0.240 [0.000-0.444] | 0.500 [0.000-1.000] | 0.515 [0.333-0.676] | 0.158 [0.000-0.333] | 0.850 [0.667-1.000] |
| GN | 0.505 [0.304-0.738] | 0.333 [0.205-0.487] | 0.606 [0.544-0.677] | 0.316 [0.118-0.500] | 1.000 [1.000-1.000] | 0.212 [0.088-0.355] | 0.188 [0.062-0.333] | 1.000 [1.000-1.000] |
| KNN | 0.379 [0.118-0.644] | 0.359 [0.205-0.513] | 0.485 [0.239-0.681] | 0.242 [0.062-0.432] | 0.667 [0.200-1.000] | 0.303 [0.156-0.471] | 0.148 [0.034-0.296] | 0.833 [0.600-1.000] |
| LDA | 0.556 [0.237-0.859] | 0.821 [0.692-0.923] | 0.553 [0.438-0.750] | 0.222 [0.000-0.571] | 0.167 [0.000-0.500] | 0.939 [0.844-1.000] | 0.333 [0.000-1.000] | 0.861 [0.735-0.971] |
| LRG | 0.626 [0.282-0.904] | 0.795 [0.641-0.897] | 0.538 [0.414-0.736] | 0.200 [0.000-0.500] | 0.167 [0.000-0.500] | 0.909 [0.794-1.000] | 0.250 [0.000-1.000] | 0.857 [0.735-0.970] |
| RF | 0.556 [0.247-0.871] | 0.256 [0.128-0.385] | 0.561 [0.514-0.618] | 0.293 [0.111-0.468] | 1.000 [1.000-1.000] | 0.121 [0.029-0.235] | 0.171 [0.059-0.306] | 1.000 [1.000-1.000] |
| SVM | 0.692 [0.473-0.888] | 0.590 [0.436-0.744] | 0.621 [0.374-0.829] | 0.333 [0.091-0.560] | 0.667 [0.200-1.000] | 0.576 [0.414-0.735] | 0.222 [0.053-0.429] | 0.905 [0.750-1.000] |
| XGB | 0.556 [0.247-0.847] | 0.795 [0.667-0.923] | 0.538 [0.417-0.723] | 0.200 [0.000-0.533] | 0.167 [0.000-0.500] | 0.909 [0.794-1.000] | 0.250 [0.000-1.000] | 0.857 [0.735-0.970] |

**Note:** Performance metrics are presented as the AUC with 95% confidence intervals in square brackets. Abbreviations: AB = Adaptive Boosting, BN = Bernoulli NB, DT = Decision Tree, GB = Gradient Boosting, GN = Gaussian NB, K Nearest Neighbors = KNN, Linear Discriminant Analysis = LDA, Logistic Regression = LRG, Random Forest = RF, SVM = Support Vector Machine, XGB = XG Boost, AUC = Area Under the Curve, NPV = Negative Predictive Value, PPV = Positive Predictive Value.

**Table S13. Performance of the pathological models on external test set.**

| Model | AUC | Accuracy | Balanced Accuracy | F1-score | Sensitivity | Specificity | PPV | NPV |
| --- | --- | --- | --- | --- | --- | --- | --- | --- |
| AB | 0.361 [0.286-0.431] | 0.591 [0.455-0.727] | 0.361 [0.286-0.431] | 0.000 [0.000-0.000] | 0.000 [0.000-0.000] | 0.722 [0.571-0.861] | 0.000 [0.000-0.000] | 0.765 [0.618-0.897] |
| BN | 0.365 [0.162-0.597] | 0.250 [0.136-0.386] | 0.396 [0.190-0.585] | 0.233 [0.056-0.391] | 0.625 [0.250-1.000] | 0.167 [0.057-0.300] | 0.143 [0.032-0.258] | 0.667 [0.333-1.000] |
| DT | 0.333 [0.171-0.519] | 0.386 [0.250-0.523] | 0.333 [0.170-0.506] | 0.129 [0.000-0.294] | 0.250 [0.000-0.571] | 0.417 [0.257-0.583] | 0.087 [0.000-0.217] | 0.714 [0.500-0.905] |
| GB | 0.319 [0.096-0.585] | 0.341 [0.205-0.477] | 0.306 [0.146-0.507] | 0.121 [0.000-0.278] | 0.250 [0.000-0.600] | 0.361 [0.206-0.514] | 0.080 [0.000-0.200] | 0.684 [0.471-0.895] |
| GN | 0.417 [0.106-0.701] | 0.318 [0.182-0.477] | 0.389 [0.174-0.588] | 0.211 [0.051-0.391] | 0.500 [0.100-0.857] | 0.278 [0.143-0.441] | 0.133 [0.029-0.281] | 0.714 [0.455-0.923] |
| KNN | 0.582 [0.343-0.811] | 0.455 [0.318-0.614] | 0.472 [0.268-0.676] | 0.250 [0.065-0.452] | 0.500 [0.143-0.875] | 0.444 [0.286-0.600] | 0.167 [0.040-0.333] | 0.800 [0.611-0.957] |
| LDA | 0.569 [0.324-0.812] | 0.773 [0.659-0.886] | 0.569 [0.424-0.762] | 0.286 [0.000-0.571] | 0.250 [0.000-0.625] | 0.889 [0.784-0.974] | 0.333 [0.000-0.750] | 0.842 [0.718-0.949] |
| LRG | 0.319 [0.149-0.526] | 0.727 [0.591-0.864] | 0.444 [0.390-0.487] | 0.000 [0.000-0.000] | 0.000 [0.000-0.000] | 0.889 [0.780-0.974] | 0.000 [0.000-0.000] | 0.800 [0.675-0.925] |
| RF | 0.236 [0.088-0.425] | 0.250 [0.136-0.386] | 0.396 [0.197-0.579] | 0.233 [0.054-0.400] | 0.625 [0.250-1.000] | 0.167 [0.054-0.297] | 0.143 [0.030-0.265] | 0.667 [0.333-1.000] |
| SVM | 0.576 [0.270-0.880] | 0.568 [0.432-0.705] | 0.542 [0.333-0.756] | 0.296 [0.062-0.514] | 0.500 [0.100-0.857] | 0.583 [0.425-0.732] | 0.211 [0.037-0.412] | 0.840 [0.667-0.964] |
| XGB | 0.562 [0.326-0.795] | 0.750 [0.636-0.864] | 0.556 [0.408-0.736] | 0.267 [0.000-0.545] | 0.250 [0.000-0.600] | 0.861 [0.750-0.971] | 0.286 [0.000-0.667] | 0.838 [0.714-0.949] |

**Note:** Performance metrics are presented as the AUC with 95% confidence intervals in square brackets. Abbreviations: AB = Adaptive Boosting, BN = Bernoulli NB, DT = Decision Tree, GB = Gradient Boosting, GN = Gaussian NB, K Nearest Neighbors = KNN, Linear Discriminant Analysis = LDA, Logistic Regression = LRG, Random Forest = RF, SVM = Support Vector Machine, XGB = XG Boost, AUC = Area Under the Curve, NPV = Negative Predictive Value, PPV = Positive Predictive Value.

**Table S14. Prediction performance of Rad-Path model compared with single-modality models**

| Model | Training set | Internal test set | External test set |
| --- | --- | --- | --- |
| **Rad-Path model** | | | |
| AUC | 1.000 [1.000-1.000] | 0.851 [0.722-0.963] | 0.814 [0.678-0.936] |
| Sensitivity | 1.000 [1.000-1.000] | 0.500 [0.000-1.000] | 0.625 [0.286-1.000] |
| Specificity | 1.000 [1.000-1.000] | 0.848 [0.714-0.969] | 0.778 [0.639-0.914] |
| PPV | 1.000 [1.000-1.000] | 0.375 [0.000-0.800] | 0.385 [0.143-0.643] |
| NPV | 1.000 [1.000-1.000] | 0.903 [0.778-1.000] | 0.903 [0.786-1.000] |
| **Tumor-region radiomics model** | | | |
| AUC | 0.821 [0.743-0.894] | 0.780 [0.529-0.964] | 0.658 [0.415-0.871] |
| Sensitivity | 0.579 [0.438-0.750] | 0.500 [0.000-1.000] | 0.375 [0.000-0.750] |
| Specificity | 0.856 [0.787-0.920] | 0.848 [0.727-0.967] | 0.722 [0.571-0.861] |
| PPV | 0.595 [0.429-0.757] | 0.375 [0.000-0.750] | 0.231 [0.000-0.462] |
| NPV | 0.848 [0.782-0.917] | 0.903 [0.778-1.000] | 0.839 [0.700-0.964] |
| **Tumor-region radiomics model** | | | |
| AUC | 0.794 [0.726-0.863] | 0.707 [0.533-0.885] | 0.538 [0.329-0.736] |
| Sensitivity | 0.553 [0.412-0.725] | 0.333 [0.000-0.750] | 0.250 [0.000-0.600] |
| Specificity | 0.923 [0.870-0.971] | 0.879 [0.743-0.971] | 0.778 [0.641-0.906] |
| PPV | 0.724 [0.571-0.875] | 0.333 [0.000-0.750] | 0.200 [0.000-0.500] |
| NPV | 0.850 [0.783-0.915] | 0.879 [0.750-0.971] | 0.824 [0.694-0.941] |
| **Pathological Titan model** | | | |
| AUC | 0.984 [0.960-0.999] | 0.692 [0.454-0.879] | 0.576 [0.255-0.851] |
| Sensitivity | 0.921 [0.824-1.000] | 0.667 [0.200-1.000] | 0.500 [0.125-0.875] |
| Specificity | 0.962 [0.919-0.991] | 0.576 [0.406-0.742] | 0.583 [0.424-0.750] |
| PPV | 0.897 [0.795-0.977] | 0.222 [0.053-0.429] | 0.211 [0.048-0.400] |
| NPV | 0.971 [0.933-1.000] | 0.905 [0.762-1.000] | 0.840 [0.696-0.962] |

**Note:** Performance metrics are presented as the AUC with 95% confidence intervals in square brackets. Abbreviations: AUC = Area Under the Curve, NPV = Negative Predictive Value, PPV = Positive Predictive Value.

**Table S15. Evaluation of patients with high and low model scores (N = 6)**

| Variable | High score 1 | High score 2 | | High score 3 | Low score 1 | | Low score 2 | Low score 3 |
| --- | --- | --- | --- | --- | --- | --- | --- | --- |
| **SubTME evaluation** | | |  | | |  |  |  |
| Pathologist 1 | Activated-dominant | Activated-dominant | | Activated-dominant | Deserted-dominant | | Deserted-dominant | Deserted-dominant |
| Pathologist 2 | Activated-dominant | Activated-dominant | | Activated-dominant | Deserted-dominant | | Deserted-dominant | Deserted-dominant |
| Pathologist 3 | Deserted-dominant | Activated-dominant | | Activated-dominant | Deserted-dominant | | Deserted-dominant | Deserted-dominant |
| Pathologist 4 | Activated-dominant | Activated-dominant | | Activated-dominant | Deserted-dominant | | Deserted-dominant | Deserted-dominant |
| **Rad-Path score** | 0.62 | 0.56 | | 0.52 | 0.04 | | 0.16 | 0.18 |
| **Predicted ER** | yes | yes | | yes | no | | no | no |
| **ER** | no | yes | | yes | no | | no | no |
| **Adjuvant chemotherapy** | GS | AG | | FOLFIRINOX | S-1 | | FOLFIRINOX | GS |

**Note:**  ER, early recurrence; Rad-Path, radiomics-pathology; SubTME, subtypes of tumor microenvironment; GS, gemcitabine plus S-1; AG, nab-paclitaxel plus gemcitabine; FOLFIRINOX, 5-fluorouracil, leucovorin, irinotecan, and oxaliplatin.

**Table S1****6 CT scanning sequences and parameters in institution 1.**

| **Parameter** | **SOMATOM Definition Flash** | **Discovery CT750 HD** | **SOMATOM Force** | **Aquilion ONE** | **IQon - Spectral CT** |
| --- | --- | --- | --- | --- | --- |
| **Kilovoltage peak (kVp)** | 100-120 | 120 | 110 | 100 | 120 |
| **Dose reference (mAs)** | Auto-adaptive | | | | |
| **Slice thickness (mm)** | 1 | 0.625 | 1 | 1 | 1 |
| **Rotation time (s)** | 0.5 | 0.6 | 0.5 | 0.275 | 0.27 |
| **Matrix** | 512×512 | | | | |
| **Collimation (mm)** | 0.60 | 0.625 | 0.60 | 0.5 | 0.625 |
| **Pitch** | 0.70, 0.90 | 0.98 | 0.60 | 0.81 | 0.98 |

**Table S17 CT scanning sequences and parameters in institution 2.**

| **Parameter** | **iCT 256** | **Revolution Ace** | **Revolution** **Apex** | **Revolution CT** | **SOMATOM Force** | **uCT780** |
| --- | --- | --- | --- | --- | --- | --- |
| **Kilovoltage peak (kVp)** | 120 | 120 | 120 | 110 | 100 | 120 |
| **Dose reference (mAs)** |  | Auto-adaptive | | | | |
| **Slice thickness (mm)** | 0.625 | 1.25 | 1.25 | 1.25 | 1 | 1 |
| **Rotation time (s)** | 0.4 | 0.8 | 0.35 | 0.35 | 0.5 | 0.5 |
| **Matrix** |  | 512×512 | | | | |
| **Collimation (mm)** | 0.625 | 0.625 | 0.625 | 0.625 | 0.6 | 0.5 |
| **Pitch** | 0.64 | 0.98 | 0.99 | 0.99 | 0.6 | 0.99 |


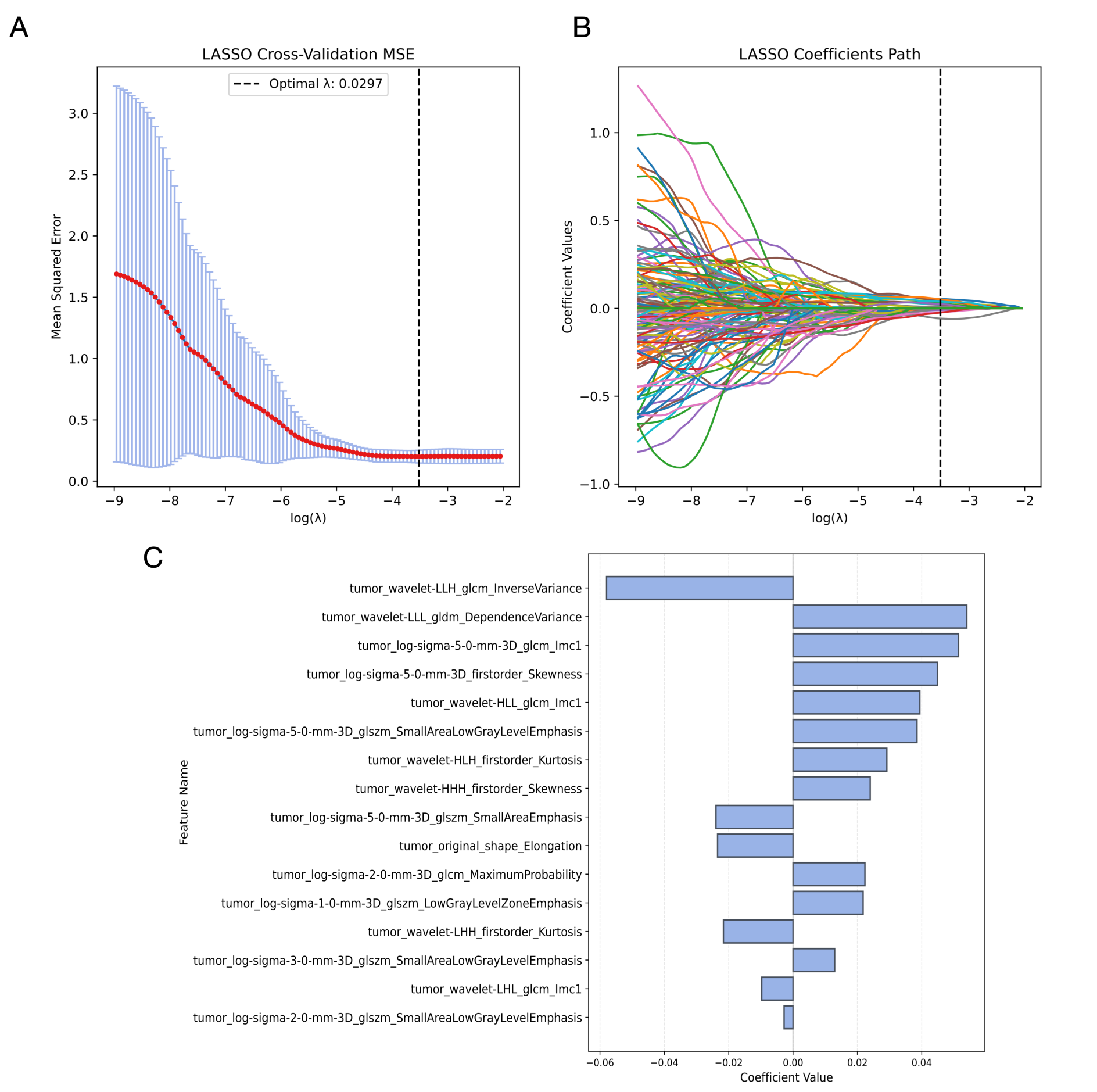


**Figure S1：**Multivariable selection of tumor-region radiomic features using LASSO regression. Using a significance threshold of P < 0.10, 361 of the 1218 tumor-region radiomic features were retained for multivariable selection. (A) Trend of the mean square error (MSE) across different values of the penalty parameter λ during cross-validation. The red dots indicate the average MSE values, with blue error bars representing the standard deviation. The optimal λ value, where the MSE is minimized, is marked by the black dotted line. (B) Convergence paths of feature weight coefficients as λ varies. Each line corresponds to a radiomic feature previously identified in univariable analysis. At the optimal λ = 0.0297, the MSE reaches its minimum value of 0.2001 ± 0.0518, and 16 features with non-zero weight coefficients are retained. (C) Names and weight coefficients of the 16 selected features.


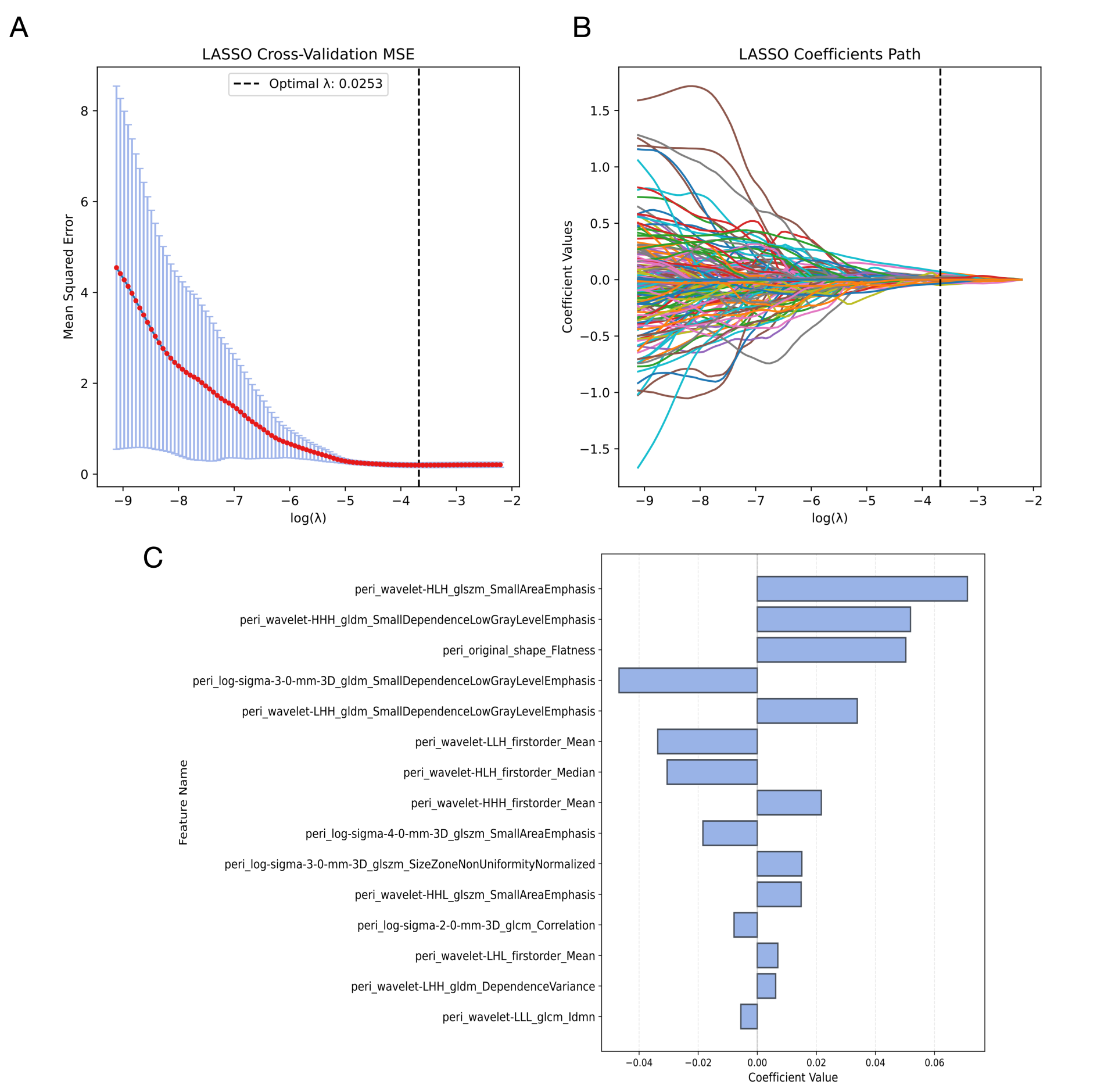


**Figure S2：**Multivariable selection of peritumoral-region radiomic features using LASSO regression. Using a significance threshold of P < 0.10, 392 of the 1218 peritumoral-region radiomic features were retained for multivariable selection. (A) Trend of the mean square error (MSE) across different values of the penalty parameter λ during cross-validation. The red dots indicate the average MSE values, with blue error bars representing the standard deviation. The optimal λ value, where the MSE is minimized, is marked by the black dotted line. (B) Convergence paths of feature weight coefficients as λ varies. Each line corresponds to a radiomic feature previously identified in univariable analysis. At the optimal λ = 0.0253, the MSE reaches its minimum value of 0.1968 ± 0.0530, and 15 features with non-zero weight coefficients are retained. (C) Names and weight coefficients of the 15 selected features.


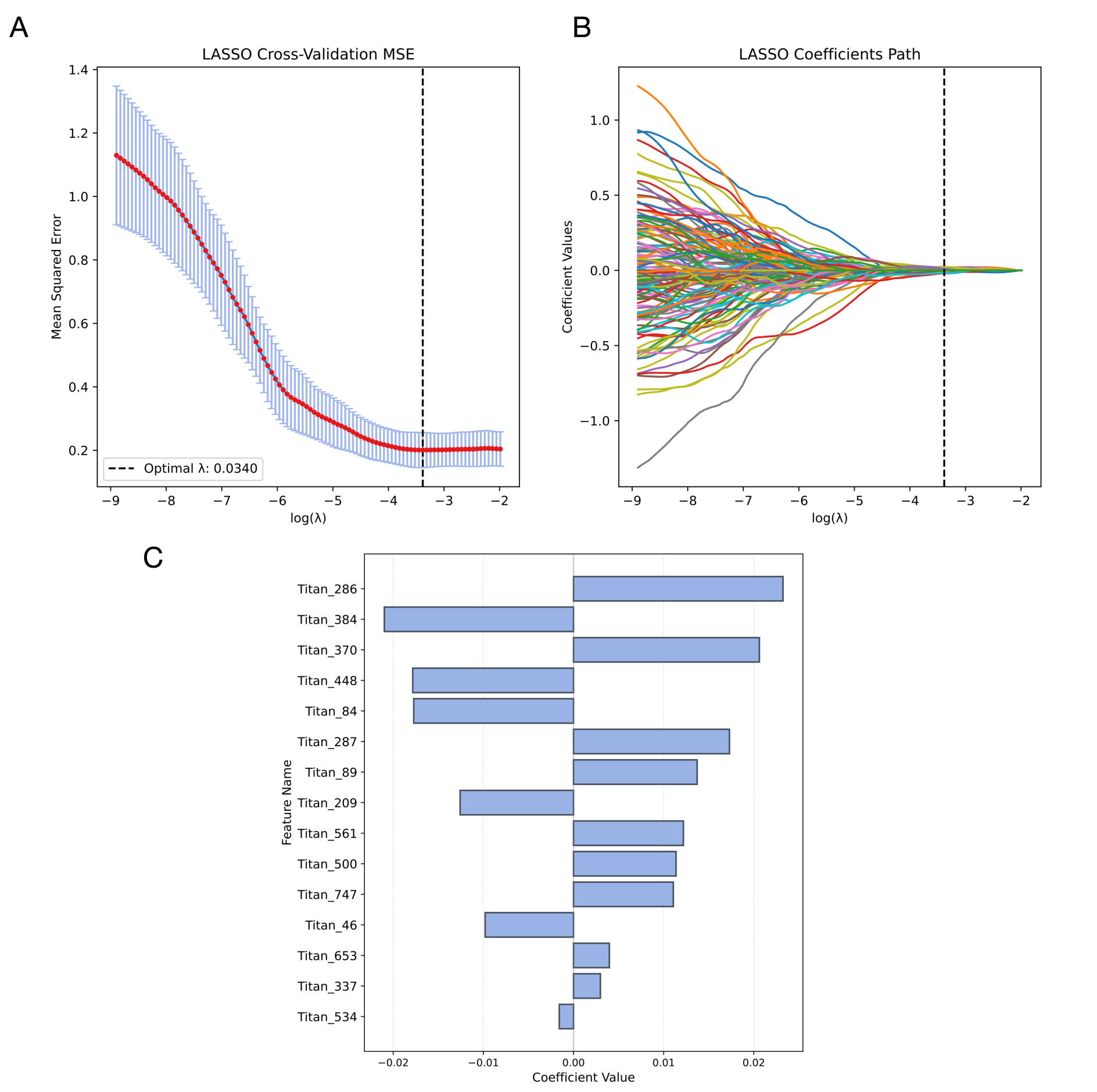


**Figure S3：**Multivariable selection of pathological Titan features using LASSO regression. Using a significance threshold of P < 0.10, 197 of the 768 pathological Titan features were retained for multivariable selection. (A) Trend of the mean square error (MSE) across different values of the penalty parameter λ during cross-validation. The red dots indicate the average MSE values, with blue error bars representing the standard deviation. The optimal λ value, where the MSE is minimized, is marked by the black dotted line. (B) Convergence paths of feature weight coefficients as λ varies. Each line corresponds to a radiomic feature previously identified in univariable analysis. At the optimal λ = 0.0253, the MSE reaches its minimum value of 0.1968 ± 0.0530, and 15 features with non-zero weight coefficients are retained. (C) Names and weight coefficients of the 15 selected features.


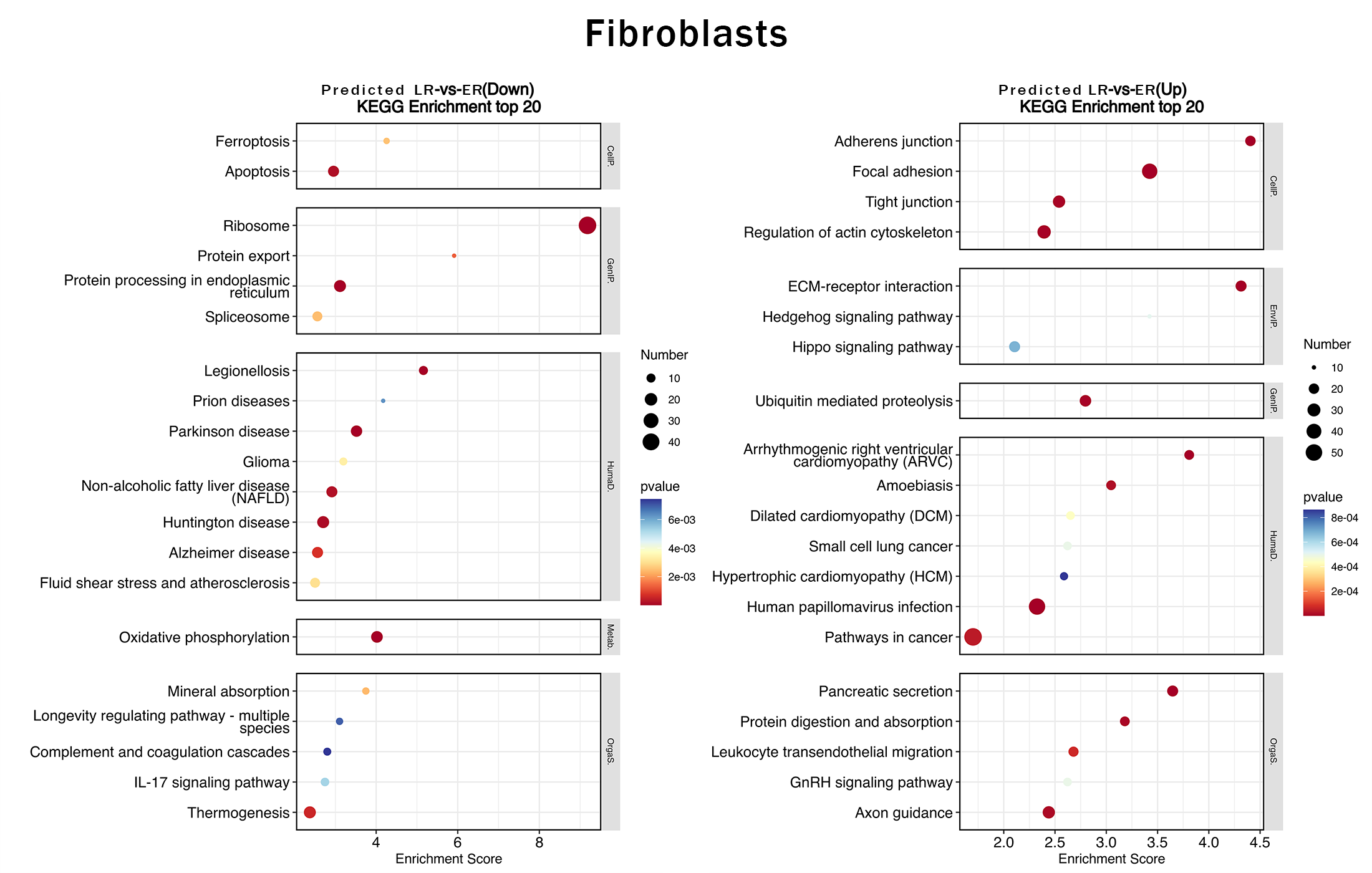


**Figure S4：**KEGG enrichment of top 20 downregulated and upregulated pathways of fibroblasts.


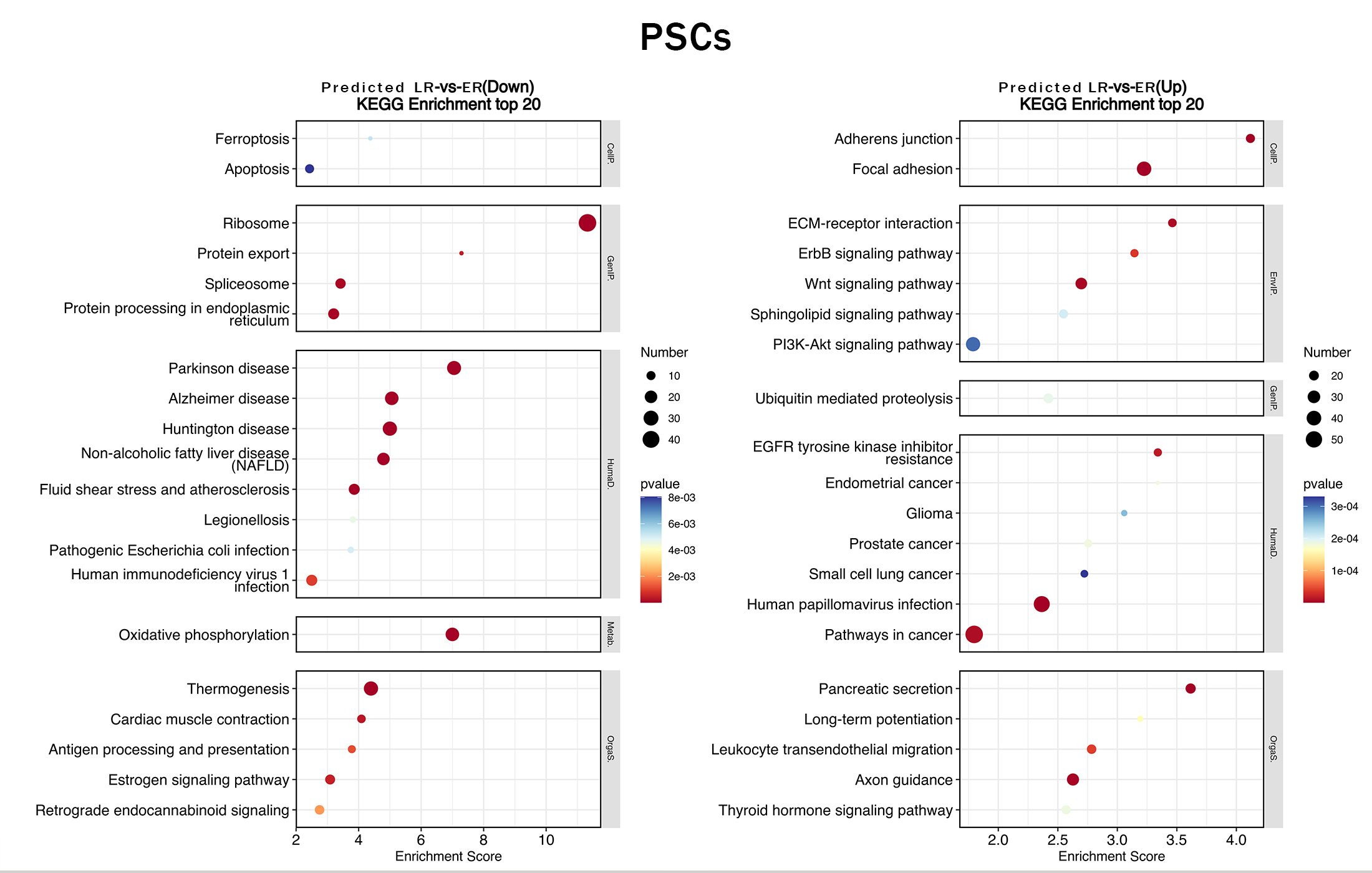
 **Figure S5：**KEGG enrichment of top 20 downregulated and upregulated pathways of pancreatic stellate cells (PSCs).


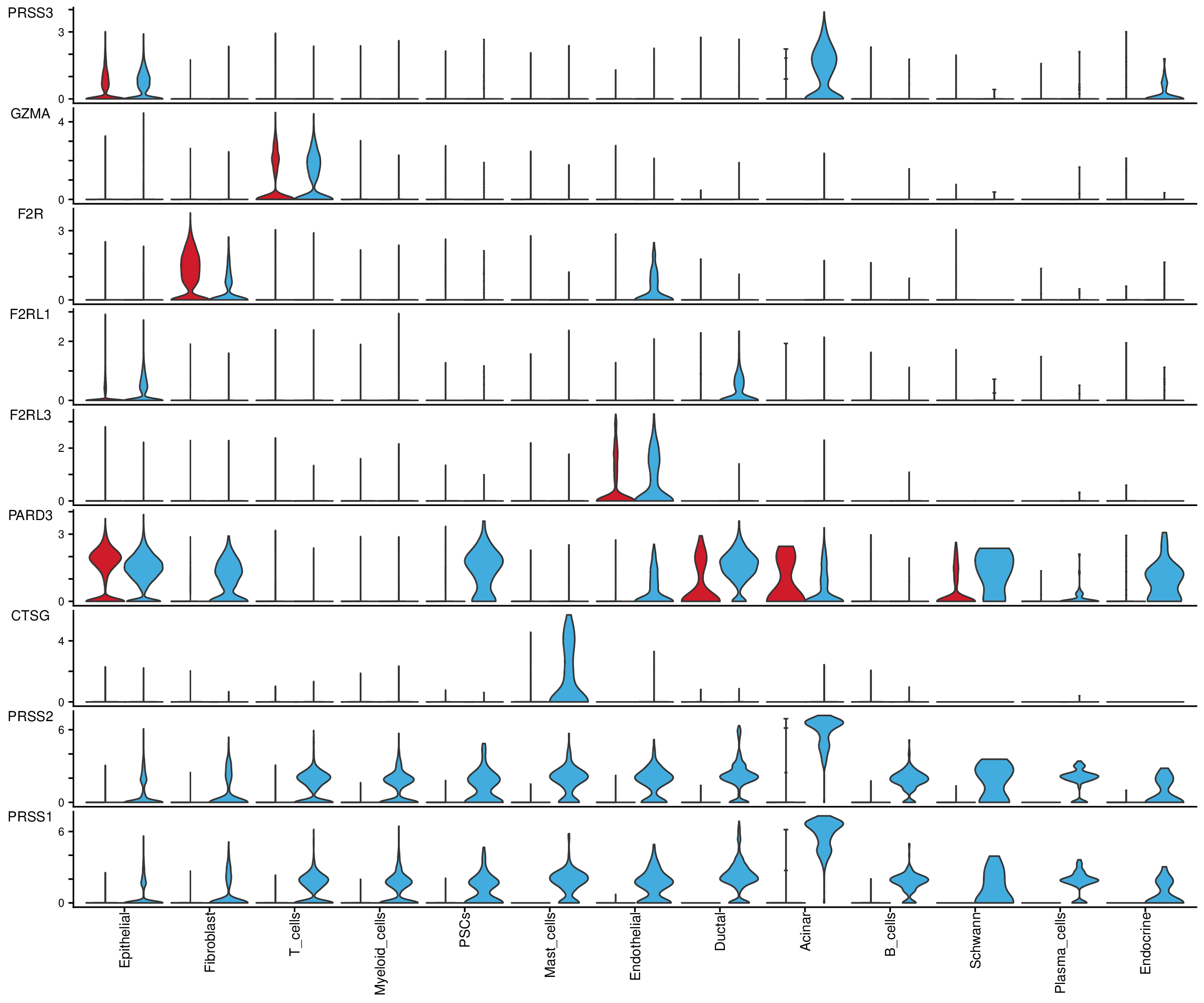
**Figure S6：**Violin plots of relative ligands and receptors between predicted ER (red) and LR (blue) groups.


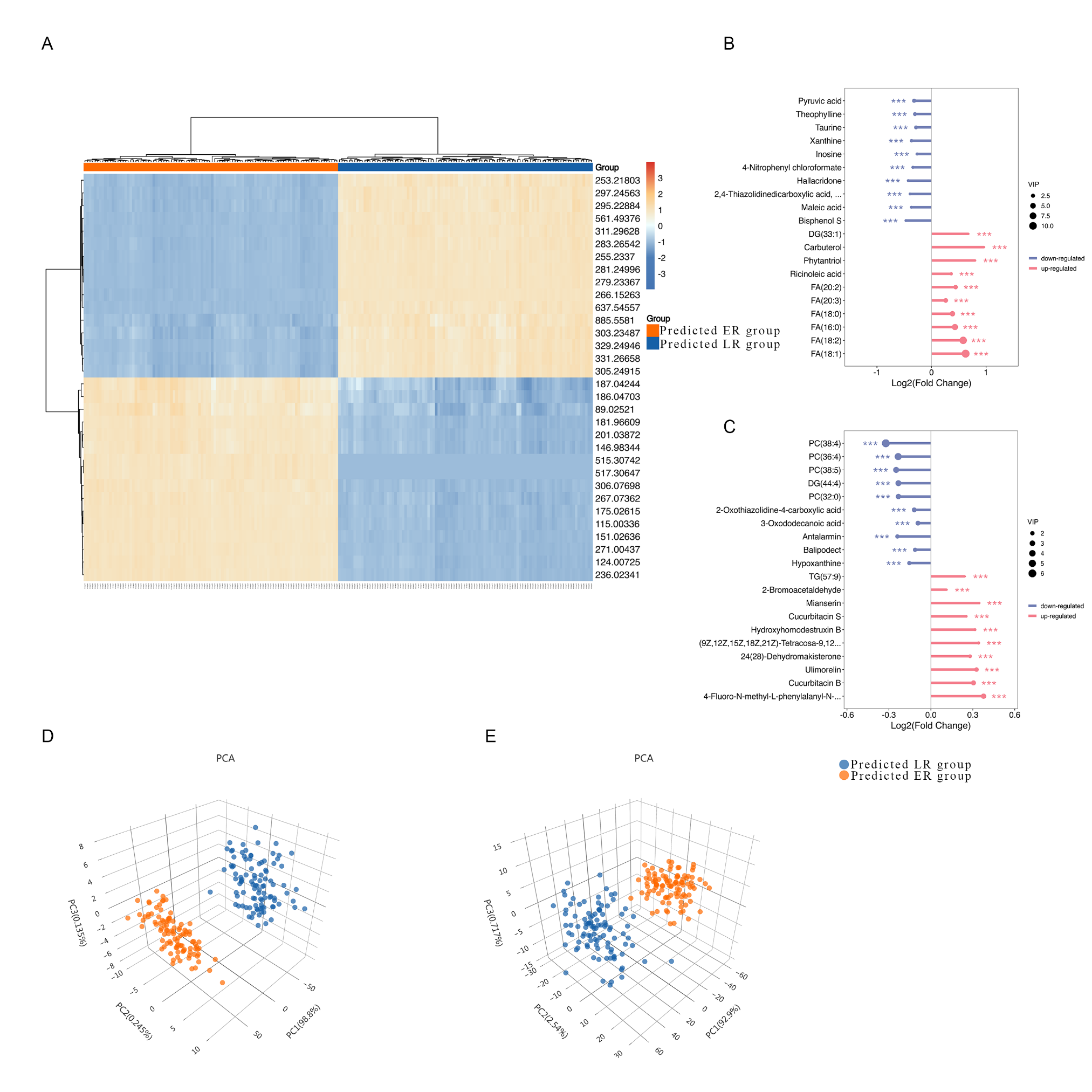
**Figure S7：**Metabolomic profiling of predicted ER and LR groups. (A) Heatmap of the top 50 differentially abundant metabolites between groups. The y-axis displays the mass-to-charge ratio corresponding to the detected molecules. (B, C) Lollipop plots of the 20 most distinctive metabolite clusters acquired in negative (B) and positive (C) ion modes. Metabolites upregulated in the predicted LR group are shown in red, and those downregulated are in blue. (D, E) Principal component analysis (PCA) score plots derived from negative (D) and positive (E) ion mode data, showing a clear separation between the predicted ER and LR groups.


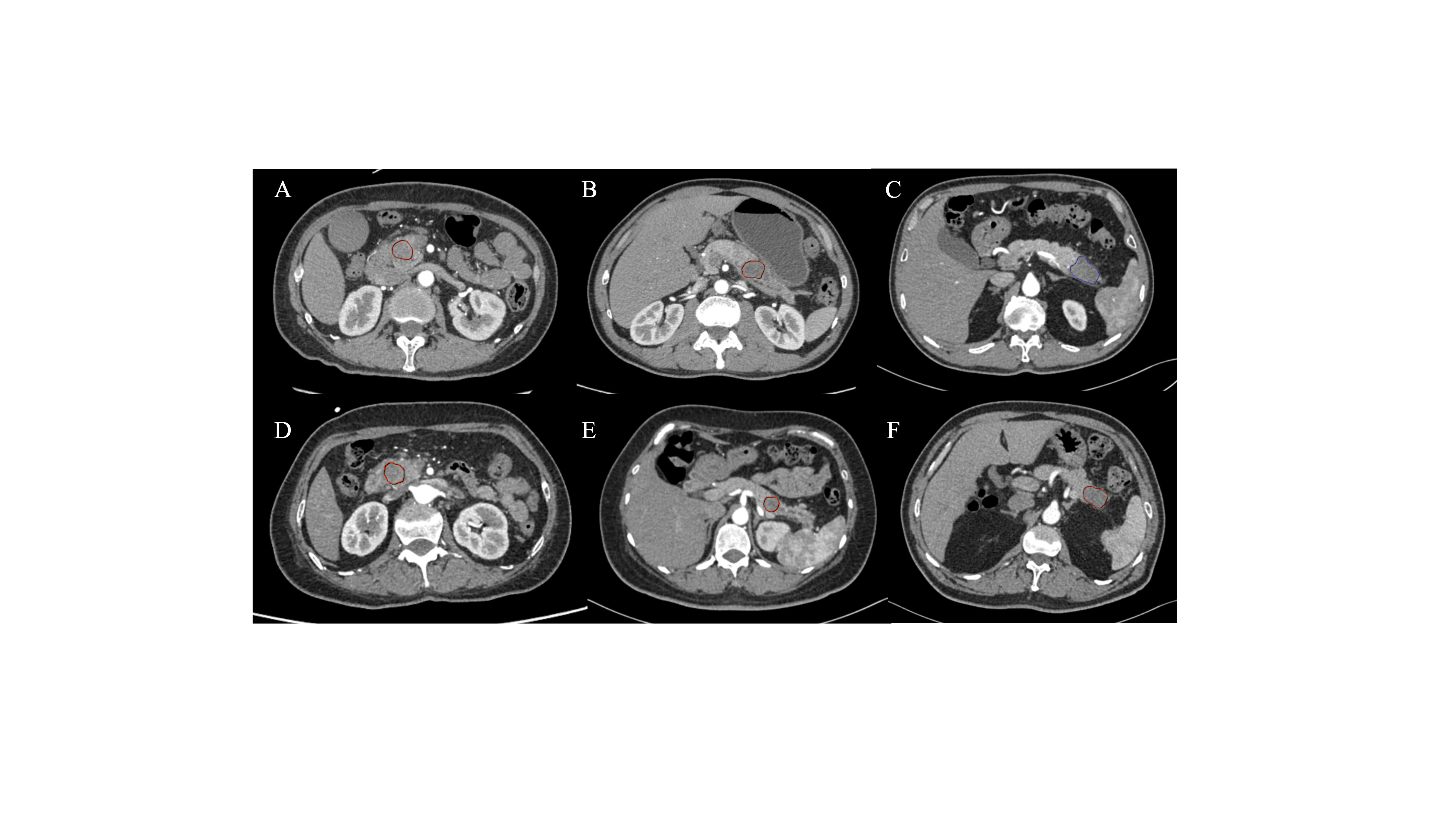


**Figure S8：**Representative CT images illustrating the clinical applicability of the Rad-Path model. (A-C) show cases from the early recurrence (ER) group, (D–F) show cases from the late recurrence (LR) group. Despite these cases exhibiting similar visual imaging appearances on contrast-enhanced CT, they were correctly predicted as ER or LR by the integrated Rad-Path model. These examples demonstrate the model’s ability to capture subtle features beyond visual inspection, highlighting its potential for precision diagnosis and clinical utility in predicting recurrence patterns.
